# Cortactin-dependent control of Par1b-regulated epithelial cell polarity in *Helicobacter* infection

**DOI:** 10.1016/j.cellin.2024.100161

**Published:** 2024-03-05

**Authors:** Irshad Sharafutdinov, Aileen Harrer, Mathias Müsken, Klemens Rottner, Heinrich Sticht, Christian Täger, Michael Naumann, Nicole Tegtmeyer, Steffen Backert

**Affiliations:** a Department of Biology, Division of Microbiology, Friedrich-Alexander-Universität Erlangen-Nürnberg, D-91058, Erlangen, Germany; b Central Facility for Microscopy, Helmholtz Centre for Infection Research, D-38124, Braunschweig, Germany; c Department of Cell Biology, Helmholtz Centre for Infection Research, D-38124, Braunschweig, Germany; d Division of Molecular Cell Biology, Zoological Institute, Technische Universität Braunschweig, D-38106, Braunschweig, Germany; e Division of Bioinformatics, Institute of Biochemistry, Friedrich-Alexander-Universität Erlangen-Nürnberg, D-91054, Erlangen, Germany; f Otto von Guericke University, Institute of Experimental Internal Medicine, Medical Faculty, D-39120, Magdeburg, Germany

**Keywords:** CagA, T4SS, Cortactin, Par1b, ZO-1, ASPP2, Tight junction, Cell polarity

## Abstract

Cell polarity is crucial for gastric mucosal barrier integrity and mainly regulated by polarity-regulating kinase partitioning-defective 1b (Par1b). During infection, the carcinogen *Helicobacter pylori* hijacks Par1b via the bacterial oncoprotein CagA leading to loss of cell polarity, but the precise molecular mechanism is not fully clear. Here we discovered a novel function of the actin-binding protein cortactin in regulating Par1b, which forms a complex with cortactin and the tight junction protein zona occludens-1 (ZO-1). We found that serine phosphorylation at S405/418 and the SH3 domain of cortactin are important for its interaction with both Par1b and ZO-1. Cortactin knockout cells displayed disturbed Par1b cellular localization and exhibited morphological abnormalities that largely compromised transepithelial electrical resistance, epithelial cell polarity, and apical microvilli. *H. pylori* infection promoted cortactin/Par1b/ZO-1 abnormal interactions in the tight junctions in a CagA-dependent manner. Infection of human gastric organoid-derived mucosoids supported these observations. We therefore hypothesize that CagA disrupts gastric epithelial cell polarity by hijacking cortactin, and thus Par1b and ZO-1, suggesting a new signaling pathway for the development of gastric cancer by *Helicobacter*.

## Highlights

• Cortactin controls Par1b-mediated cell polarity and defines cellular and nuclear architecture
• Cortactin SH3 domain and S405/S418 phosphorylation promote Par1b/ZO-1 interactions
• *H. pylori* CagA stimulates cortactin interaction with the tight junction protein ZO-1
• *H. pylori* promotes cortactin/Par1b/ZO-1 interactions in a CagA-dependent manner
• *H. pylori* infection affects host cell polarity in human-derived gastric mucosoids

## Graphical abstract

**Figure:**
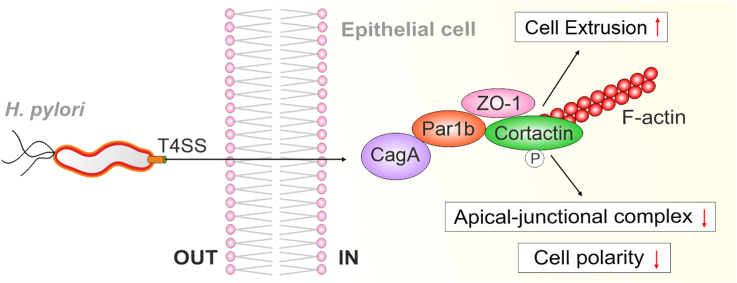

## 1 Introduction

Tightly regulated cell polarity is compulsory for establishment of accurately structured epithelia and functioning of multicellular organisms (Buckley & St Johnston, 2022). Specialized proteins located in apical, lateral and basal regions of epithelial cells provide proper intercellular interactions and accordingly adapt cytoskeletal dynamics. Transmembrane proteins such as occludin, claudins and E-cadherin form cellular junctions, bringing neighboring cells together and regulating paracellular transport of metabolites (Zihni et al., 2016). Located in the cytoplasm, zonula occludens (ZO)-1, -2, -3 proteins and catenins connect apical junctional proteins with cytoskeletal components. On the other hand, three major complexes maintain cell polarity, the partitioning defective (Par) complex, the Crumbs complex, and the Scribble complex (Ruch & Engel, 2017). The Crumbs complex along with atypical protein kinase C (aPKC), Par6, and the Rho GTPase Cdc42 define the apical cell membrane. Par3 targets to apical junctions, while the Scribble complex and Par1b localize at the basolateral side. Moreover, actin microfilaments, microtubules and intermediate filaments form the core of the cytoskeleton that along with other regulating proteins provides mechanical forces to maintain cell shape, polarity and a variety of motile functions (Moujaber & Stochaj, 2020). One of the major cytoskeletal components, actin, dynamically assembles into polymeric filamentous (F)-actin from monomeric globular (G)-actin and *vice versa*. In a traditional view, the major actin nucleation machines, the formins and the Arp2/3 complex, mostly promote actin polymerization into arrays harboring linear and branched filaments, respectively (Gautreau et al., 2022). However, whereas the branching activity of Arp2/3 complex is mediated by a family of nucleation promoting factors (NPFs) known as Wiskott-Aldrich syndrome protein (WASP)-family members, Arp2/3 complex activation by WISH/DIP/SPIN90 (WDS)-family proteins has recently also appeared to generate linear, unbranched actin filaments (Cao et al., 2023). Yet another class of proteins, previously referred to as class II-NPFs, including the most prominent member cortactin, its hematopoietic counterpart HS1 and Abp1 mainly stabilize Arp2/3 complex-containing branch junctions, adding to the complexity of biochemical states and cellular functions exerted by Arp2/3 complex (Gautreau et al., 2022, Guo et al., 2018; Liu et al., 2024, Cao & Way, 2024).

Cortactin is an actin-binding protein capable of interacting with the Arp2/3 complex and actin filaments via its N-terminal acidic (NTA) and a more central F-actin binding domain, respectively. Furthermore, cortactin contains an alpha-helical domain followed by a proline-rich region (PRR) and a C-terminal Src homology 3 (SH3) domain that provide interactions with multiple other proteins, resulting in tight regulation of actin-cytoskeletal functions (Schnoor et al., 2018; Weaver, 2008). Early work proposed cortactin to function as NPF, but cortactin alone activates the Arp2/3 complex only weakly, and rather plays a major role in branched actin network stabilization (Liu et al., 2024), and in multiplying the nucleation activity of canonical NPFs, i.e. neuronal WASP (N-WASP) (Fregoso et al., 2023; Martinez-Quiles et al., 2004). Various functions of cortactin are regulated by post-translational modification events e.g., phosphorylation or acetylation (Schnoor et al., 2018; Sharafutdinov et al., 2022c). Serine phosphorylation of cortactin at S113, S405 and S418 by p21-associated kinase (PAK) and extracellular regulated 1–2 (Erk1/2) kinases, and tyrosine phosphorylation at Y421, Y470 and Y486 by Src, Syk, Fer and Abl kinases control cortactin's interaction partners and downstream signaling. Inhibitory studies have pinpointed a major role for cortactin in vital cellular activities related to cellular endocytosis, adhesion and migration (Ammer & Weed, 2008). Importantly, cortactin gene (*cttn*) amplification and protein overexpression are associated with carcinogenesis of various etiology, including gastric cancer (Wei et al., 2014; Yin et al., 2017). Due to its aforementioned roles, various microbial pathogens target cortactin to manipulate cytoskeleton activities (Selbach & Backert, 2005; Sharafutdinov et al., 2022c). Numerous intracellular pathogens are reported to hijack cortactin signaling to promote their invasion into host cells, e.g. *Shigella flexneri*, *Listeria monocytogenes* and *Rickettsia conorii*. Multiple other studies have found a distinct role for cortactin during infection by the gastric pathogen *Helicobacter pylori* (Sharafutdinov et al., 2020). *H. pylori* is the first described bacterial carcinogen and utilizes the unique effector molecule CagA delivered into host cells by the type IV secretion system (T4SS) composed of structural VirB1-VirB11 and some accessory proteins (Fischer et al., 2020). For this purpose, *H. pylori* establishes intimate contact with various host cell receptors, e.g. integrins and CEACAMs (Javaheri et al., 2017; Kwok et al., 2007; Moonens et al., 2018). Once injected, CagA triggers a series of signaling events that manipulate multiple cellular factors including cortactin (Tegtmeyer et al., 2011). For example, injected CagA inactivates Src, which triggers tyrosine dephosphorylation and simultaneously serine phosphorylation of cortactin at S405 and S418 by Erk1/2 kinases (Selbach et al., 2003). This leads to cortactin interaction with and activation of focal adhesion kinase (FAK) that enhances cell adhesion, thus preventing excessive host cell lifting during *H. pylori* infection. Another established role of cortactin during *H. pylori* infection is maintaining sustained CagA phosphorylation via activation of FAK, Src and Abl kinases (Knorr et al., 2021).

*H. pylori* is a prominent cause of various gastric disorders ranging from gastritis, gastric and duodenal ulcers to gastric adenocarcinoma and mucosa-associated lymphoid tissue (MALT) lymphoma (Salama et al., 2013). Notably, injected CagA induces the overexpression of cortactin by a JNK-dependent pathway, suggesting a role in *H. pylori*-associated gastric cancer development (Sharafutdinov et al., 2021). Aside from this, CagA was shown to interact with the partitioning kinase Par1b that resulted in disruption of cell polarity through Par1b inactivation (Saadat et al., 2007). In particular, transfection of CagA into polarized Madin-Darby canine kidney (MDCK) monolayers resulted in mislocalization of Par1b, E-cadherin and gp135, followed by extrusion of CagA-expressing cells (Saadat et al., 2007). Additionally, CagA targets the apoptosis-stimulating protein of p53 2 (ASPP2), and was proposed to be involved in the subversion of cell polarity complex proteins such as Par3 and aPKC (Buti et al., 2011, 2020). The CagA-mediated disruption of cell polarity was anticipated to constitute one of the key events contributing to gastric cancer development. However, additional, potentially involved factors, and the subcellular location of CagA-induced disruption of Par1b-mediated cell polarity have remained unclear. Here we provide a completely new function of cortactin that mediates targeting of Par1b to ZO-1, which helps *H. pylori* to inactivate Par1b, followed by cell lifting of CagA-positive cells.

## 2 Results

### 2.1 Cortactin controls cellular architecture and cell polarity through Par1b

**Fig.1:**
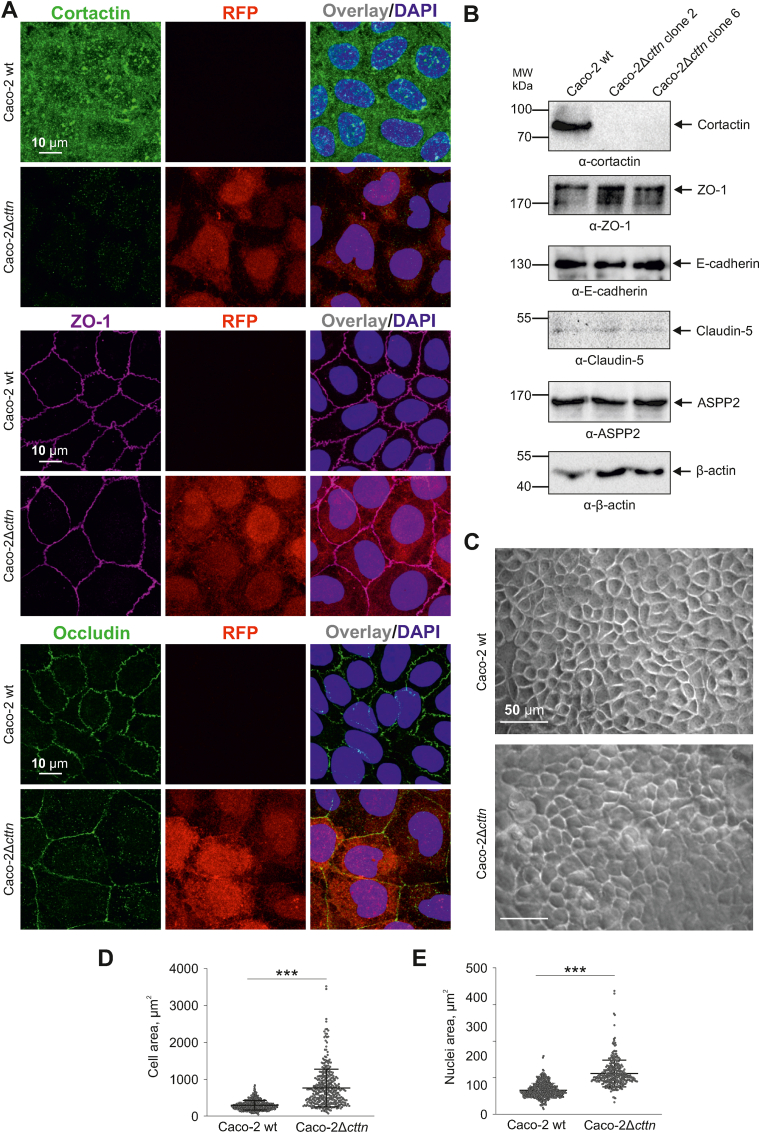
**Characterization of cortactin gene knockout in intestinal Caco-2 cells by CRISPR-Cas9.** (**A**) Confirmation of cortactin gene knockout in Caco-2Δ*cttn* cells by fluorescence microscopy using cortactin-specific antibodies (green) and detecting the functional HDR plasmid, expressing RFP (red). Immunostaining of ZO-1 and occludin served as controls. (**B**) Western blot showing a ∼85 kDa protein corresponding to cortactin in Caco-2 wt cells, but not in Caco-2Δ*cttn* mutant clones 2 and 6. Western blotting with antibodies specific for ZO-1, E-cadherin, Claudin-5, ASPP2 and β-actin served as control. (**C**) Phase-contrast microscopy of representative Caco-2 wt and Caco-2Δ*cttn* cell lines showing cellular morphologies within respective monolayer. Quantification of cell (**D**) and nuclear (**E**) areas based on F-actin and nuclear staining, respectively, displaying arithmetic means ± SD (standard deviation) as well as individual values (dots). The size differences between Caco-2 wt and Caco-2Δ*cttn* cells were confirmed to be statistically significant with *p* ​< ​0.001 (∗∗∗).

**Fig.2:**
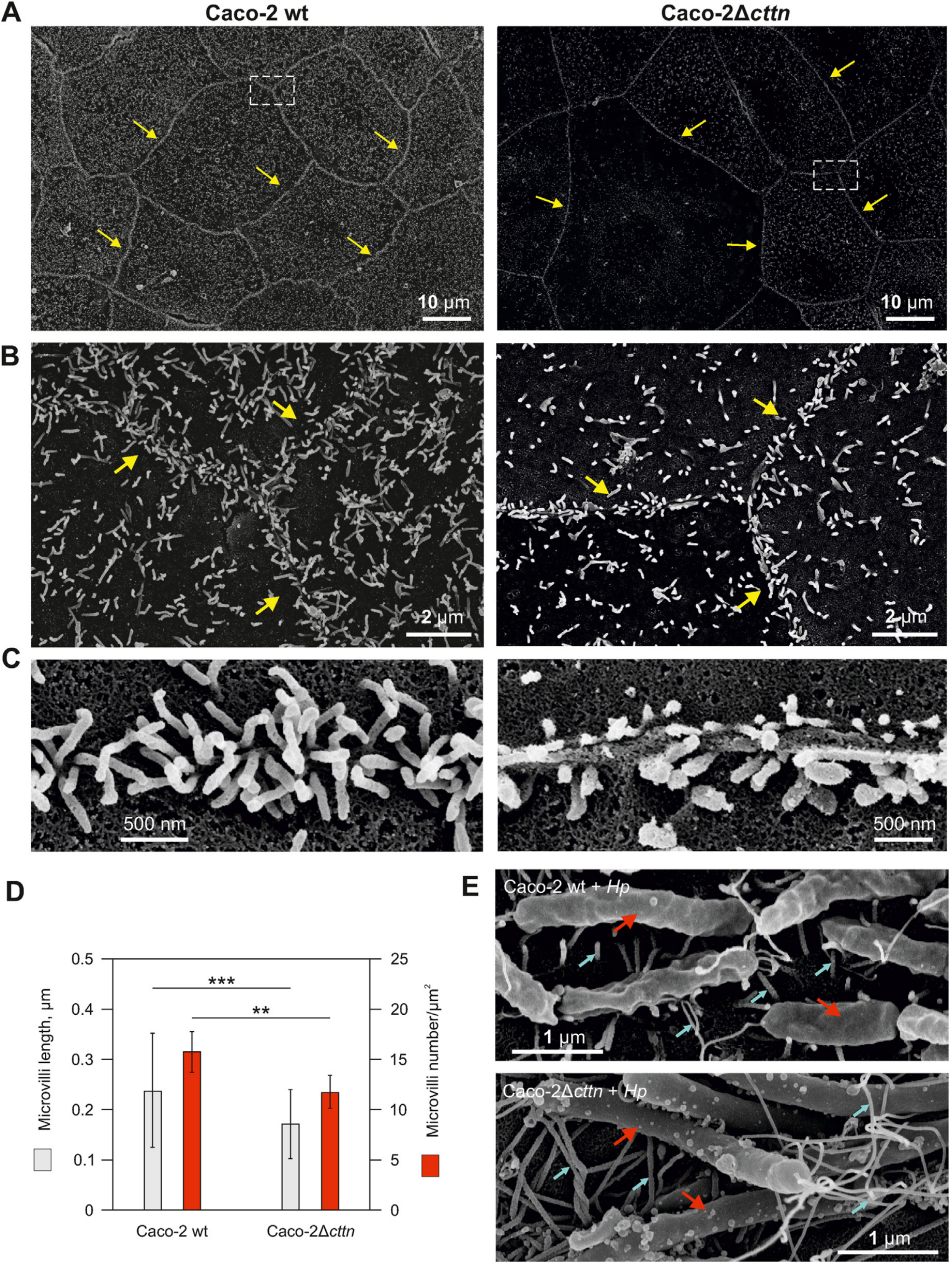
**Apical-junctional microvilli show impaired development in cortactin-deficient cells.** Scanning electron microscopy (SEM) of Caco-2 wt and Caco-2Δ*cttn* cell monolayers (**A**), triple cellular junctions (**B**), or enlarged bicellular junctions (**C**). White rectangles in panel (**A**) indicate the respective areas of triple junctions enlarged on panels shown in **B**. Yellow arrows indicate bicellular junctions. (**D**) The microvilli number and their length were quantified and expressed as mean values ​± ​SD; *p* ​< ​0.001 (∗∗∗). (**E**) SEM of *H. pylori*-infected Caco-2 cells, demonstrating similar bacterial adherence levels onto both wt and Δ*cttn* knockout cells. Red arrows mark the bacterial bodies, and blue arrows show either microvilli or flagellae.

Cortactin is a major regulator of actin cytoskeleton dynamics and a key target of microbial pathogens. In particular, *H. pylori* was reported to hijack cortactin function to control host cell dynamics (Tegtmeyer et al., 2011). Importantly, *H. pylori* was also shown to disrupt host cell polarity via targeting Par1b (Saadat et al., 2007), however, it has remained unknown whether cortactin is implicated in this process. To study this in more detail, we established clonal cell lines stably disrupted for the cortactin gene in the polarized intestinal epithelial cell line Caco-2 using CRISPR/Cas9 (Caco-2 *cttn* knockout cells, Δ*cttn*). Altogether, we selected 8 single Δ*cttn* clones. Two Δ*cttn* clones (2 and 6) were used for further detailed characterization and gave similar results. Both Caco-2 wild-type (wt) and Δ*cttn* cell lines expressed comparable levels of junctional proteins ZO-1, occludin, E-cadherin, Claudin-5 and ASPP2 (Fig. 1A and B, Fig. S1A). Phase contrast microscopy revealed enlarged cell size of *cttn* knockout compared to wt cells (Fig. 1C), suggesting disturbed signaling to cytoskeletal architecture. In accordance with these results, fluorescence microscopy of the actin cytoskeleton and DAPI staining revealed significantly enlarged cell areas of Caco-2Δ*cttn* cells, as well as enlarged nuclei (Fig. 1D and E; Fig. S1B). Interestingly, Caco-2 wt monolayers exhibited uniform development and distribution of regular microvilli structures across the cells, while Δ*cttn* cells showed impaired microvilli formation characterized by reduced quantity and shorter length (Fig. 2A–C; Figs. S2A and B). The number and length of microvilli structures near tight junctions (TJs) were significantly decreased in Δ*cttn* cells (Fig. 2D; Figs. S2C–D). However, cellular colonization of *H. pylori* during infection was not compromised by *cttn* knockout (Fig. 2E; Fig. S3).

**Fig.3:**
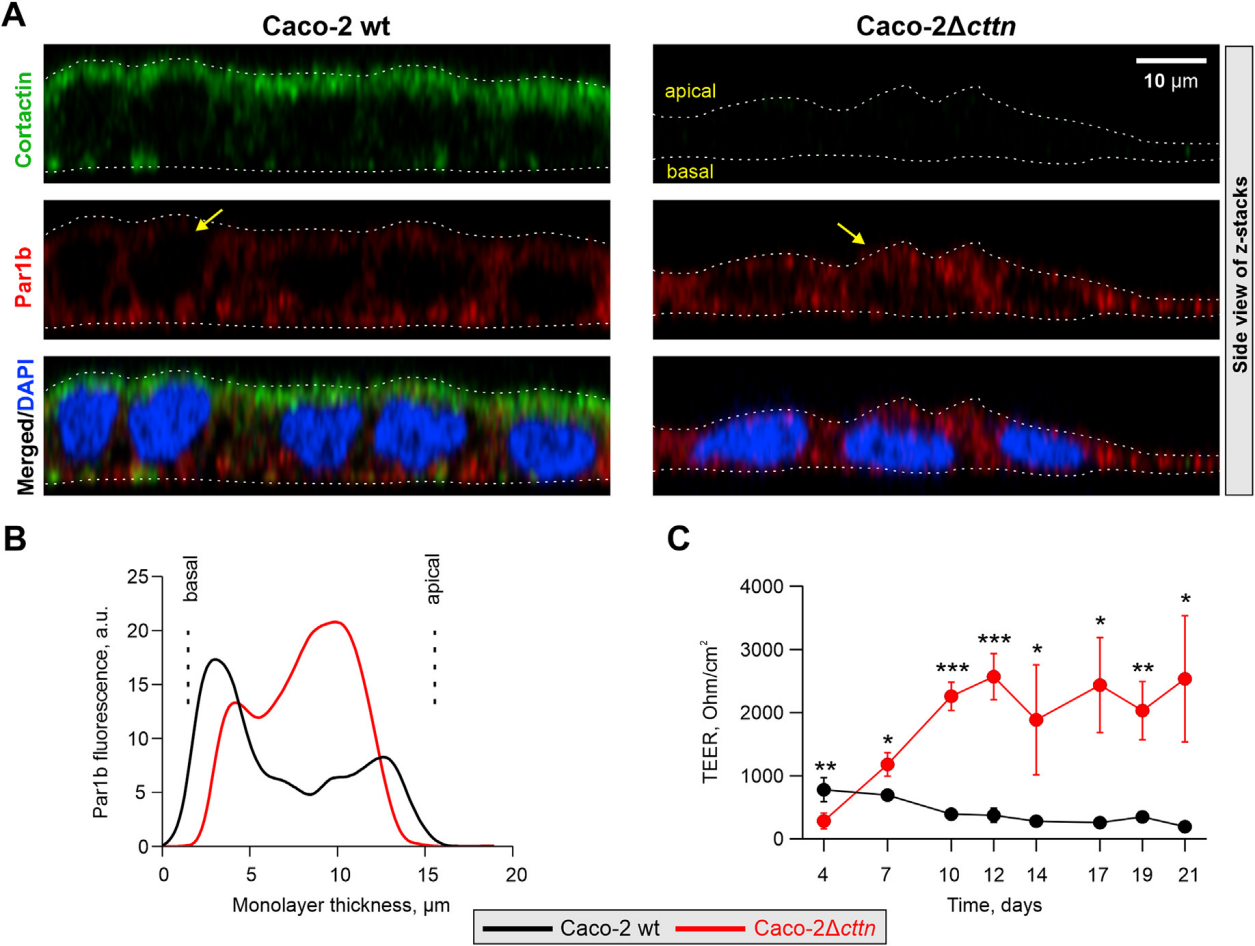
**Cortactin deficiency results in anomalous cellular morphology and defective Par1b-mediated cell polarity in Caco-2 monolayers**. (**A**) Fluorescence microscopy of the Caco-2 monolayer cross-sections (X/Z dimension). The white dashed lines indicate apical and basal cell surfaces. (**B**) The Par1b fluorescence Z-profiles of representative cells (indicated by yellow arrows) are plotted to show the distribution of Par1b in basal/apical dimension; a.u. – arbitrary units. (**C**) TEER of Caco-2 wt and Caco-2Δ*cttn* was measured for 21 days showing increased TEER values in the knockout cells. The mean TEER values ± SD in Ohm/cm^2^ are presented; statistical significances are shown as *p* ​< ​0.05 (∗), *p* ​< ​0.01 (∗∗) and *p* ​< ​0.001 (∗∗∗). The two-way ANOVA analysis revealed statistically significant differences between the two cell lines (*p*<0.001) and the growth time points (*p*<0.01). Further analysis showed an interaction effect between cell line (Caco-2 wt or Δ*cttn*) and growth time on TEER values (*p*<0.001).

We next immunostained Caco-2 cells with α-cortactin and α-Par1b antibodies, and counterstained nuclei with DAPI. Confocal microscopy and further analyses confirmed the flattened morphology of Δ*cttn* cell monolayers (Fig. 3A; Fig. S4A). Quantification of Par1b fluorescence in apical-basal dimensions (Z-profile) displayed Par1b mislocalization from basal into apical sides (Fig. 3B), indicating disturbed cell polarity in Δ*cttn* cells. In addition, the overall length of bilateral junctions were reduced (by almost 50%) in Δ*cttn* monolayers (Figs. S4B–C). Interestingly, transepithelial electrical resistance (TEER) significantly increased in Δ*cttn* knockout compared to wt cell monolayers (Fig. 3C), which suggests a particular yet unknown role for cortactin in regulating cell-cell contacts and barrier functions. Of note, this is at variance to previously published data using RNAi-mediated *cttn* knockdown (Citalan-Madrid et al., 2017), which largely focused on shorter periods of cultivation than analyzed here. In contrast, we have generated cell lines with permanent *cttn* disruption, and propose that continuous lack of cortactin triggers subcellular repositioning of Par1b, which may be directly targeted by cortactin for the control of proper cell polarity in wt cells.

### 2.2 Cortactin SH3 domain and serine phosphorylation at S405/S418 are crucial for the interaction with Par1b and ZO-1

**Fig.4:**
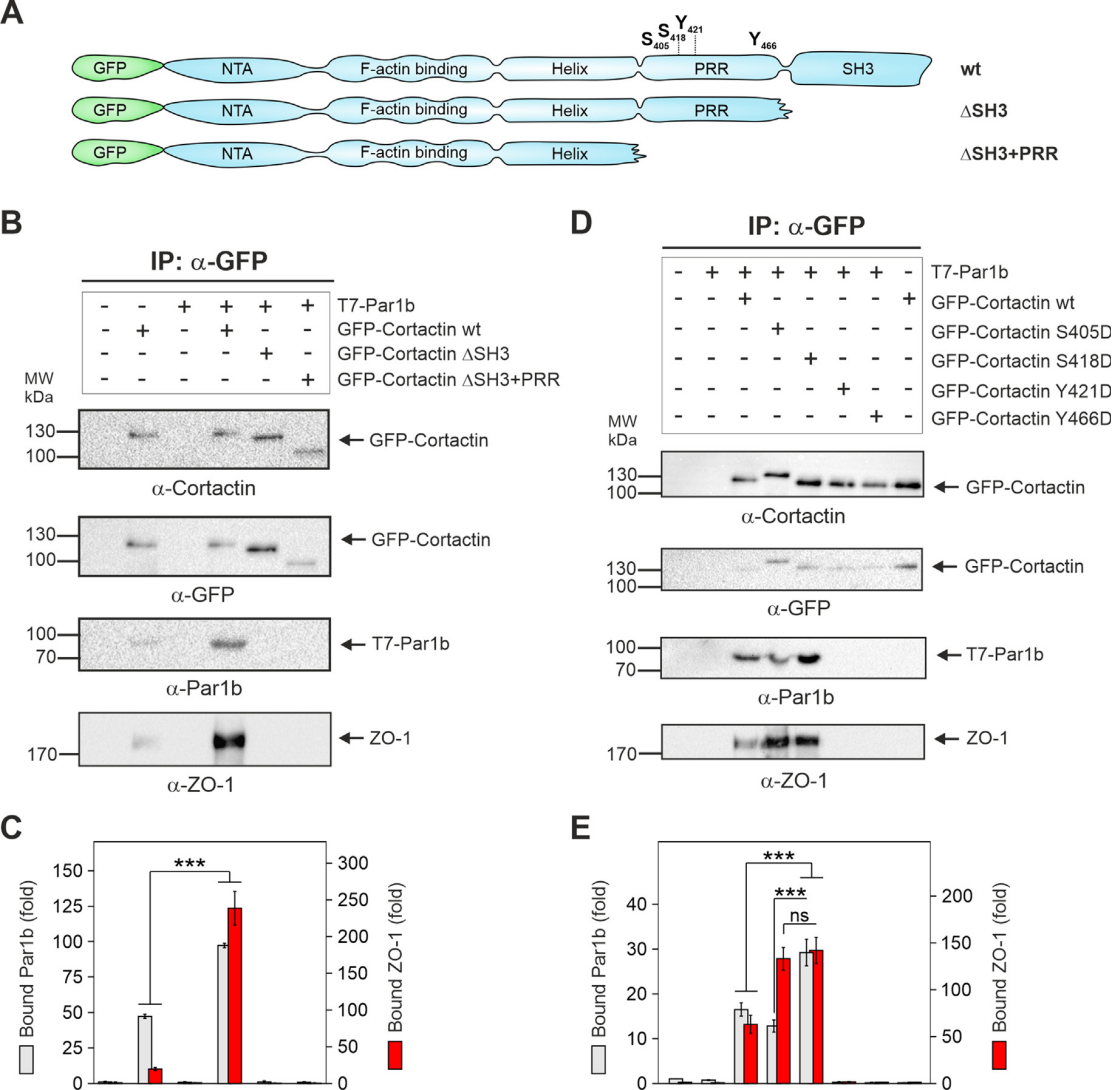
**Cortactin SH3 domain and phosphorylation at S405/S418 are required for efficient complex formation with Par1b and ZO-1**. (**A**) Schematic illustration of the cortactin constructs used to study the interaction with the partner proteins Par1b and ZO-1. (**B**) AGS cells were transfected with the T7-Par1b construct and co-transfected with wt or truncated cortactin constructs. After 48 h, samples were subjected to immunoprecipitation (IP) with α-GFP antibodies, and analyzed by Western blotting using antibodies against cortactin, GFP, Par1b and ZO-1, as indicated. (**C**) The band intensities of Par1b and ZO-1 immunoprecipitated with GFP-cortactin were quantified and expressed as complex-bound proteins. The mean intensities ​± ​SDs are presented; *p* ​< ​0.001 (∗∗∗). (**D**) AGS cells were transfected with various GFP-cortactin constructs possessing phosphorylation-mimetic point-mutations S405D, S418D, Y421D and Y466D, and co-transfected with the T7-Par1b construct for 48 h. Samples were subjected to IPs with α-GFP antibodies and analyzed using Western blotting with indicated antibodies. (**E**) Band intensities of Par1b and ZO-1 immunoprecipitated with GFP-cortactin were quantified and expressed as relative, protein complex-bound Par1b or ZO-1. The mean intensities ​± ​SDs are presented; *p* ​< ​0.001 (∗∗∗).

Since cortactin-deficient Caco-2 cells displayed disturbed Par1b localization, we hypothesized that both proteins might interact with each other. A multifaceted regulation of cortactin involves its manifold interacting partners and resulting cellular activities (Schnoor et al., 2018). In particular, serine and/or tyrosine phosphorylation of cortactin in the PRR controls a number of downstream signaling pathways, while the SH3 domain facilitates interactions with other proteins. To investigate if the PRR and/or SH3 domains of cortactin are involved in binding of Par1b or ZO-1, we co-transfected cells with various T7 epitope-tagged Par1b and GFP-cortactin wt and mutant constructs (Fig. 4A). Immunoprecipitation (IP) with α-GFP antibodies and Western blot analysis indicated that expression of wt cortactin resulted in a strong complex formation with exogenously expressed Par1b and endogenous ZO-1 (Fig. 4B–C). Interestingly, only a weak ZO-1 binding was observed in the absence of exogenously expressed Par1b. Presumably, Par1b is necessary for ZO-1 binding to cortactin, potentially via regulation of ZO-1 or cortactin conformation. Deletion of either SH3 or SH3/PRR domains in cortactin largely diminished the interactions with both Par1b and ZO-1 (Fig. 4B–C). Taking together, the IP analysis indicates that the SH3 domain of cortactin promotes the interactions with Par1b and ZO-1.

It was previously shown that cortactin phosphorylated at serine residues 405 and/or 418 mediates SH3-dependent binding to signaling factors (Martinez-Quiles et al., 2004; Tegtmeyer et al., 2011). In particular, *H. pylori* infection was shown to hijack cortactin function via the SH3 domain and S405/S418 phosphorylation (Tegtmeyer et al., 2011). We therefore transfected cells with GFP-cortactin constructs harboring phosphorylation-mimetic point mutations (S405D, S418D, Y421D and Y466D), and co-transfected them with a T7-Par1b plasmid. IPs using anti-GFP antibodies followed by Western blot analysis revealed that cortactin mutants S405D and S418D displayed the strongest interactions with exogenously expressed Par1b and endogenous ZO-1 (Fig. 4D–E). In contrast, tyrosine phospho-mimetic cortactin variants (Y421D or Y466D) displayed abolished interaction with either Par1b or ZO-1. These results suggest that serine-phosphorylation of cortactin at residues S405 and/or S418 drives the interactions with Par1b and ZO-1, and *H. pylori* infection could possibly disturb cell polarity via these interactions.

### 2.3 *H. pylori* CagA promotes cortactin interaction with the tight junction protein ZO-1

**Fig.5:**
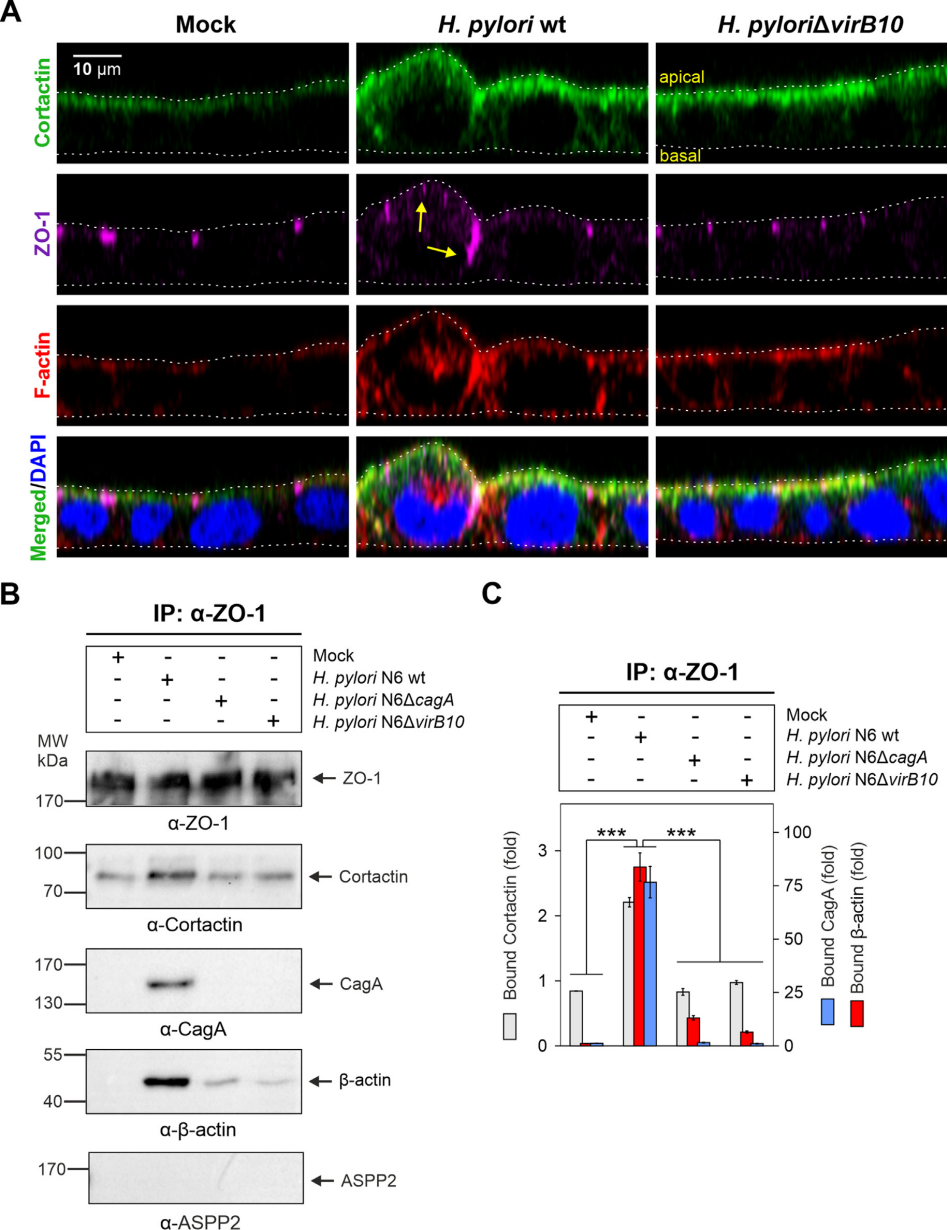
***H. pylori* CagA promotes complex formation of cortactin with ZO-1**. (**A**) Fluorescence microscopy of Caco-2 wt cell monolayer cross-sections (X/Z dimension) without or after infection with *H. pylori* wt or Δ*virB10* mutant. The white dashed lines indicate apical and basal surfaces of the monolayers. Yellow arrows indicate abnormal localization of ZO-1. (**B**) Western blot analysis of protein complex formation in Caco-2 wt cells after *H. pylori* infection (MOI 100) for 6 h by IP using α-ZO-1 antibodies. Proteins co-immunoprecipitated with ZO-1 were probed using antibodies against cortactin, CagA, β-actin and ASPP2. (**C**) The band intensities of cortactin, β-actin and CagA immunoprecipitated in a complex with ZO-1 were quantified and expressed as relative, protein complex-bound cortactin, β-actin or CagA. Mean intensities ​± ​SD are presented; *p* ​< ​0.001 (∗∗∗).

Since cortactin knockout affects cellular junctions and TEER, we proposed that this may be regulated through interactions with Par1b and ZO-1. Infections with *H. pylori* were recently shown to affect the phosphorylation status both of cortactin (Tegtmeyer et al., 2011, 2021) and Par1b (Saadat et al., 2007) via the injected effector protein CagA. We therefore investigated if *H. pylori* affects epithelial barrier function through cortactin/Par1b interactions via ZO-1, which could potentially disturb ZO-1-mediated apical cell polarity. For this purpose, we used polarized Caco-2 cells to study either bacterial transmigration rates, TEER dynamics or protein interactions using IP and fluorescence microscopy. *H. pylori* showed slightly enhanced transmigration rates through Δ*cttn* monolayers with maximum values reached at 24 h post infection (Fig. S5A), which might be due to the reduced height of the monolayer (Fig. 3A). The TEER values were higher in Caco-2Δ*cttn* monolayers before infection (Fig. S5B). However, TEER showed a decreasing trend during the course of infection of both Caco-2 wt and Δ*cttn* cells (Fig. S5B). We next aimed to study the localization of the cortactin/ZO-1 complex within cells upon infection with *H. pylori*. We immunostained infected Caco-2 monolayers with α-cortactin and α-ZO-1 antibodies and counterstained with F-actin and DAPI. Analysis of Caco-2 cross-sections by confocal microscopy confirmed the partial co-localization of cortactin with ZO-1 (Fig. 5A, Fig. S5C). Infection with *H. pylori* wt, but not with the T4SS-deficient Δ*virB10* mutant, resulted in ZO-1 mislocalization out of the TJ areas into either apical or basolateral directions, suggesting that translocated CagA triggers ZO-1 mislocalization. This is in line with previous studies showing that CagA can alter ZO-1 localization in the apical-junctional complex (Amieva et al., 2003). Further, *H. pylori* wt led to increased cortactin binding to ZO-1, with CagA and β-actin being present in the complex, confirming aforementioned observations (Fig. 5B–C). In contrast, infection with Δ*cagA* or Δ*virB10* mutants did not induce complex formation beyond mock infection conditions, suggesting that injection of CagA is required. Furthermore, we did not detect ASPP2 within the complex of ZO-1 and CagA (Fig. 5B), implying that subversion of the cortactin-dependent apical-junctional polarity complex acts independently of the CagA/ASPP2/Par3/aPKC pathway reported previously (Buti et al., 2020).

### 2.4 *H. pylori* infection stimulates cortactin/Par1b/ZO-1 interactions in a CagA-dependent manner

**Fig.6:**
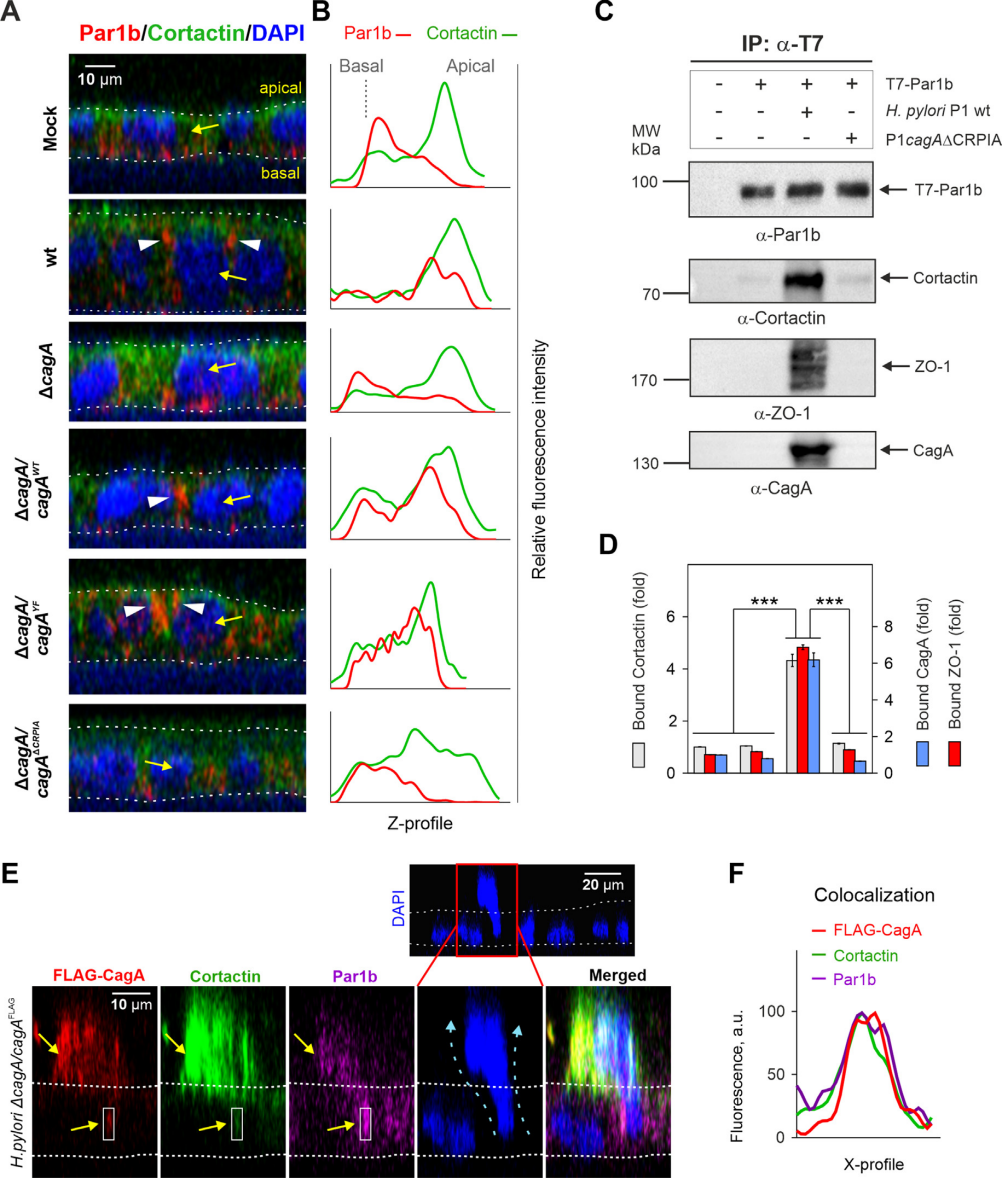
***H. pylori* CagA promotes Par1b complex formation with cortactin and ZO-1.** (**A**) Fluorescence microscopy of Caco-2 wt cell monolayer cross-sections (X/Z dimension) without or after infection with *H. pylori* wt, Δ*cagA*, Δ*cagA*/*cagA*^wt^, Δ*cagA*/*cagA*^YF^ or Δ*cagA*/*cagA*^CRPIA^ mutants. The white dashed lines indicate apical and basal surfaces of the monolayers. Short white arrowheads indicate mislocalized, apicolateral protein distribution. (**B**) Z-profiles of Par1b and cortactin fluorescence from the cell monolayer cross-section of representative cells, as indicated by yellow arrows in panel **A**, show the basal and apical distributions of proteins. (**C**) Caco-2 cells were first transfected with the T7-Par1b construct for 48 h, followed by 6 h infection with either *H. pylori* wt or the *cagAΔ*CRPIA inactivation mutant. The samples were immunoprecipitated with α-T7 antibody and analyzed by Western blotting using antibodies against T7, cortactin, ZO-1 and CagA. (**D**) Band intensities of cortactin, ZO-1 and CagA immunoprecipitated in a complex with T7-Par1b were quantified and displayed as relative, protein complex-bound cortactin, ZO-1 or CagA. Mean intensities ​± ​SDs are presented; *p* ​< ​0.001 (∗∗∗). (**E**) Enlarged images of a representative *H. pylori* wt (expressing FLAG-tagged CagA)-infected Caco-2 cell extruding from the monolayer (shown in red rectangle in the DAPI-stained monolayer). Yellow arrows indicate sites with FLAG-CagA, cortactin and Par1b co-localizing together. Blue dashed arrows show the suggested detachment route of the cell. (**F**) Colocalization analysis of FLAG-CagA, cortactin and Par1b in the cell area indicated by white rectangles in panel E. The relative fluorescence intensities of the proteins in arbitrary units (a.u.) were plotted against their spatial distribution in the X/Z-axis (X-profile).

Transfected CagA has previously been shown to interact with Par1b using the CRPIA (conserved repeats responsible for phosphorylation-independent activity)-motifs (Saadat et al., 2007). Owing to the above-mentioned cortactin-Par1b interactions, we were interested to investigate if cortactin is involved in CagA/Par1b interactions. We performed a series of infections with *H. pylori* wt or the Δ*cagA* deletion mutant as well as the complemented variants re-expressing wt, phosphorylation-resistant (YF) or CRPIA-motif-mutated CagA. Confocal microscopy revealed Par1b mislocalization from the basal to the apical side of Caco-2 cells infected with *H. pylori* harboring both wt and phosphorylation-resistant CagA (Fig. 6A; Fig. S6A). Furthermore, CRPIA-mutated CagA was unable to affect Par1b distribution. The corresponding *H. pylori* mutant resulted in a pattern comparable to the complete Δ*cagA* knockout, with Par1b distributing mainly to basolateral sides (Fig. 6A; Fig. S6A). Co-localization analyses revealed that cortactin fluorescence could be detected along with Par1b fluorescence (Fig. S6B). Interestingly, in infections with strains expressing either wt or phosphorylation-resistant CagA, Par1b/cortactin formed larger apical compartments (Fig. S6B, violet arrowheads), while CRPIA-mutated CagA or the Δ*cagA* mutant resulted in smaller Par1b/cortactin compartments at basolateral sides (Fig. S6B, blue arrowheads). Finally, Z-profiling of protein fluorescence along the basal-apical dimension confirmed Par1b re-distribution to apical sides in Caco-2 cells infected with wt or the phosphorylation-resistant, but not with CRPIA-mutated CagA (Fig. 6B).

Since ZO-1 is yet another known host protein targeted by *H. pylori* (Amieva et al., 2003; Krueger et al., 2007), we next investigated whether ZO-1 interacts with Par1b upon infection. We first transfected Caco-2 cells with the T7-Par1b construct, and then infected them with the above-described *H. pylori* strains. Probing the samples using α-phosphotyrosine antibodies indicated that CagA was properly delivered into and phosphorylated in host cells by *H. pylori* expressing wt CagA or CagAΔCRPIA (Selbach et al., 2002; Figs. S6C–D). IP with α-T7 antibodies was performed to investigate which proteins are present in the complex with Par1b. Western blot analyses revealed that infection with *H. pylori* wt induced the formation of a protein complex harboring CagA, Par1b, cortactin and ZO-1 (Fig. 6C–D). As expected, when infected with the *ΔcagA*/*cagA*^Δ*CRPIA*^ mutant, neither CagA, ZO-1, nor cortactin interacted with Par1b (Fig. 6C–D).

The aforementioned, CagA-driven cortactin/Par1b interactions and recently shown Rac1 activation (Tegtmeyer et al., 2021) suggested that disrupted cell polarity and actin cytoskeleton remodeling triggered by *H. pylori* might result in specific host cell features. To identify such changes, we infected Caco-2 monolayers with *H. pylori* wt and immunostained for cortactin and ZO-1, and counterstained for F-actin and nuclei. Using confocal microscopy, we have identified some epithelial cells extruding from the infected monolayer, which were positive for cortactin, ZO-1 and F-actin (Fig. S7A). Analysis of fluorescence intensities across the X-axis within the indicated X/Z-area (X-profile) showed a co-localization of all three proteins (Fig. S7B). Cell extrusion was previously observed in polarized MDCK monolayers transfected with an expression vector for CagA (Saadat et al., 2007). We therefore infected Caco-2 monolayers with either *H. pylori* wt (expressing FLAG-tagged CagA) or the Δ*virB10* mutant. After infection, we immunostained the cells with α-cortactin, α-Par1b and α-FLAG antibodies as well as DAPI. Intriguingly, we further observed some epithelial cells extruding from the monolayer to exhibit a strong CagA/cortactin/Par1b staining (Fig. 6E, Fig. S8), reflecting a stress-responsive force (Ohsawa et al., 2018). Nuclear DAPI staining as well as CagA/cortactin/Par1b co-staining could be observed to be positioned both within the cell monolayer and in part above it, suggesting cell extrusion. Finally, analysis of fluorescence intensities across the X-axis within the indicated X/Z-area (X-profile) showed a co-localization of CagA, cortactin and Par1b (Fig. 6F).

### 2.5 Infection affecting host cell polarity in gastric mucosoids derived from human tissue

**Fig.7:**
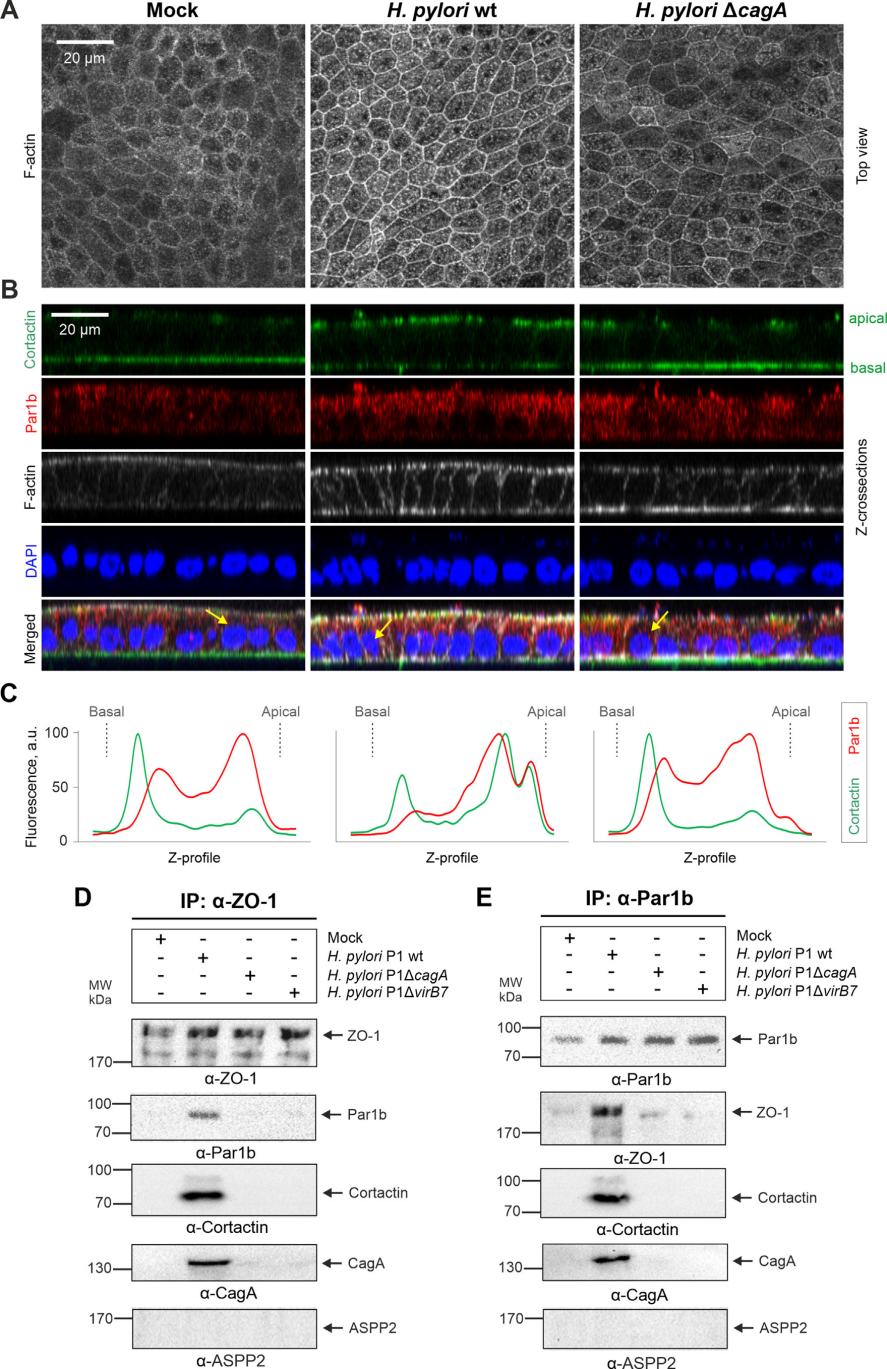
***H. pylori* deregulates the host proteins cortactin, Par1b and ZO-1 in human mucosoids via CagA.** (**A**) Fluorescence microscopy of human mucosoids after 6 h infection with either *H. pylori* wt or Δ*cagA* mutant. Polarized mucosoids were cultured in a transwell system followed by fixation in PFA and staining with phalloidin to visualize F-actin structures. (**B**) Immunofluorescence microscopy of mucosoid cross-sections (X/Z dimension) after 6 h infection with either *H. pylori* wt or Δ*cagA* mutant. (**C**) Z-profiles of Par1b, cortactin, F-actin and DAPI fluorescence from the mucosoid cross-section of representative cells (indicated by yellow arrows in panel **B**) show the basal and apical distributions of proteins; a.u. – arbitrary units (**D**-**E**) Human mucosoids were infected with *H. pylori* wt or either Δ*cagA* or Δ*virB7* mutants. The samples were immunoprecipitated with α-ZO-1 (**D**) or α-Par1b (**E**) antibodies, and analyzed by Western blotting using antibodies against ZO-1, Par1b, cortactin, CagA and ASPP2.

To study *H. pylori* infection closer to *in vivo* conditions, we next used gastric organoids derived from human tissue and grown as polarized mucosoids, an established model that provides typical morphological features of the stomach (Backert et al., 2016). Fluorescence microscopy of F-actin-stained mucosoids revealed proper monolayer formation (Fig. 7A). Surprisingly, distribution of Par1b fluorescence in gastric mucosoids was somewhat different from that observed in Caco-2 cells, with a shift towards the apical side. Infection with *H. pylori* wt, but not with the Δ*cagA* mutant, was characterized by an even more pronounced redistribution of Par1b and cortactin from basal to apical sides (Fig. 7B). Quantification of fluorescence intensity across monolayers showed the fluorescence peak of Par1b to be diminished at the basal side of mucosoids infected with *H. pylori* wt (Fig. 7C). Furthermore, cortactin was also observed to dramatically relocate from basal to apical sides in mucosoids infected with *H. pylori* wt, but not with the Δ*cagA* mutant (Fig. 7B–C). Further IP analyses of infected mucosoids with either α-ZO-1 or α-Par1b antibodies revealed CagA-dependent interactions of ZO-1, Par1b and cortactin (Fig. 7D–E). In particular, the cortactin/Par1b/ZO-1/CagA complex was immunoprecipitated from the mucosoids infected with wt *H. pylori,* but not with the Δ*cagA* or T4SS-deficient (Δ*virB7*) mutants. In line with the data shown further above, ASPP2 was again not present in the cortactin/Par1b/ZO-1/CagA complex (Fig. 7D-E, bottom). Taken together, all this data adds significant new information on *H. pylori* infections of the human gastric epithelial layer leading to the disturbance of host cell polarity by affecting the prominent host factors cortactin, Par1b and ZO-1, with a pivotal role of bacterial CagA in this process.

## 3 Discussion

**Fig.8:**
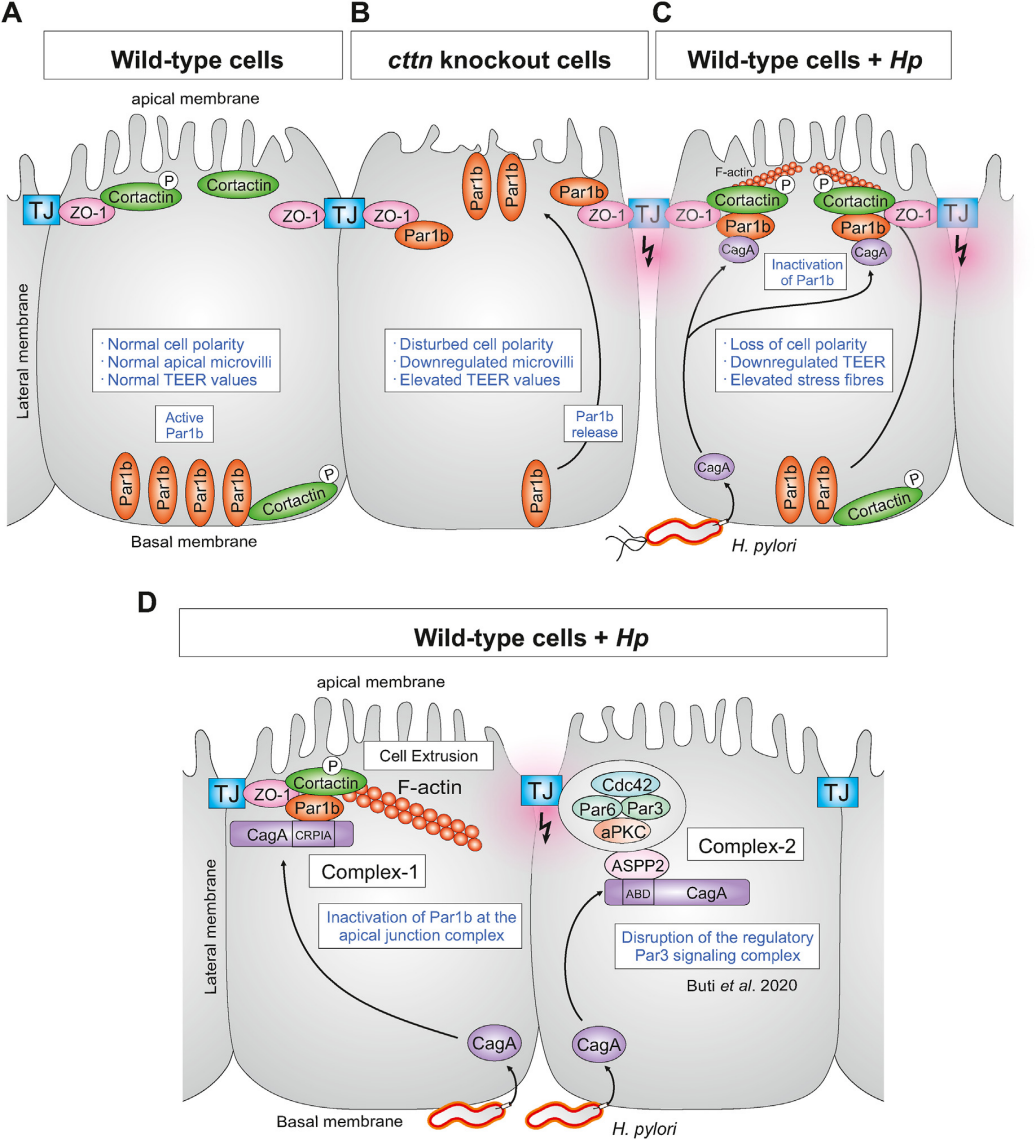
**Models highlighting the importance of cortactin in regulating cell polarity through tight junctions (TJ).** (**A**) A simplified overview shows that wild-type epithelial monolayers exhibit normal cell polarity provided directly by the Par polarity proteins and indirectly by cortactin. Par1b mainly locates to the basal membrane, as expected. In addition, cortactin seems to regulate monolayer permeability, presumably via the apical junctional complex, in particular by binding to ZO-1. This binding requires serine-phosphorylation of cortactin. (**B**) In cortactin-deficient cells, Par1b mainly locates to the apical membranes leading to disturbed cell polarity. We therefore propose that cortactin exhibits some suppressive activity on Par1b in the TJs. Thus, elevated TEER values were measured that may arise from the absence of cortactin. In addition, the expression of cortactin seems important for proper microvilli formation. (**C**) In *H. pylori*-infected wild-type monolayers, injected CagA induces Par1b inactivation and serine-phosphorylation of cortactin, associated with strongly enhanced complex formation with ZO-1 in the TJs, which leads to loss of cell polarity. (**D**) During infection with *H. pylori,* CagA is injected into epithelial cells, which targets Par1b and cell polarity by two different pathways, here named complex-1 and complex-2. In complex-1, CagA binds directly to Par1b via the CRPIA-motif, which triggers loss of cell polarity and may promote cell extrusion. This complex also contains ZO-1 and cortactin. In complex-2, CagA targets ASPP2 at the ABD (ASPP2 Binding Domain) to inhibit the aPKC-containing apical regulatory complex. In this way, aPKC cannot phosphorylate Par3 (Buti et al., 2020) and CagA-bound Par1b (Saadat et al., 2007) any longer, which abrogates their mutual antagonistic activities. We propose that both signaling complexes together result in full disruption of cell polarity and induction of cytoskeletal rearrangements.

A hallmark of epithelial cell monolayers is their polarized nature containing three major subcellular sites - an apical membrane harboring microvilli and TJs, lateral membrane surfaces connecting neighboring epithelial cells, and a basal membrane that associates with the extracellular matrix. This fundamental apical–basal polarization phenotype ensures proper function of the epithelia, such as a first barrier against intruding microbial pathogens and nutrient exchange. The TJs are composed of multiple transmembrane proteins including tricellulin, occludin and claudins being connected with the cytosolic scaffolding proteins ZO-1, ZO-2 and ZO-3, which assemble at the most apical intersection of adjacent cells (Ruch & Engel, 2017; Zihni et al., 2016). In addition, various cell polarity factors control the proper composition and function of the TJs and polarized cellular architecture. A regulatory complex comprising aPKC, Par3, Par6 and Cdc42 has been described as a major module controlling apical-junctional characteristics (Ruch & Engel, 2017; Zihni et al., 2016), as active aPKC can phosphorylate important junction and cell polarity proteins including Par1b, Par3 and others (Buckley & St Johnston, 2022). In this way, aPKC activity determines the subcellular distribution of Par1b, which localizes to basal or basolateral membranes, and is specifically excluded from the apical side (Benton & St Johnston, 2003; Böhm et al., 1997). In addition to these factors, an early report described that TJs can also contain the actin-binding protein cortactin, but its function in TJs has remained elusive (Katsube et al., 1998). Notably, interaction of cortactin and ZO-1 was proposed to play a role in colorectal cancer progression (Hirakawa et al., 2009). On the other hand, in patients with inflammatory bowel disease (IBD), decreased cortactin levels and loss of co-localization with ZO-1 were associated with intestinal epithelial barrier dysfunction (Citalan-Madrid et al., 2017). Here, we generated a cortactin knockout of the epithelial Caco-2 cell line**,** which revealed numerous surprising phenotypes such as enlarged cells with compromised apical microvilli, mis-localization of Par1b from basal to apical sides, and elevated TEER values, suggesting new functions for cortactin in cell polarity and microvilli assembly (Fig. 8A and B). While the role of cortactin in microvilli and nuclear architecture needs to be investigated in future studies, we could demonstrate that Par1b binds directly to ZO-1 of cortactin knockout cells, suggesting that the absence of cortactin prevents proper function of Par1b and even induces apical targeting of Par1b to TJs. This is remarkable and suggests the interference of cortactin with epithelial permeability, which could support the upregulation of TEER in its absence. One explanation for this phenotype might be cortactin-dependent regulation of claudin pores that provide paracellular transport of small ions and/or water (Piontek et al., 2020). Mechanistically, cortactin could regulate claudin function via the scaffold protein ZO-1, which links cellular junctions with cytoplasmic cytoskeleton components including cortactin (Katsube et al., 1998). In fact, our gene knockout of cortactin in Caco-2 cells phenotypically mimics the reduced epithelia permeability observed in polarized MDCK cells upon Claudin-2 knockout (Tokuda & Furuse, 2015). In addition, a similar relationship between cell morphology and TEER has been observed in differently cultured porcine jejunal IPEC-J2 epithelial cells (Zakrzewski et al., 2013) and human bronchial Calu-3 epithelial cells (Grainger et al., 2006), where cells with highly columnar shapes exhibited lower TEER values than cuboidal ones. Taken together, reduced epithelial permeability by cortactin knockout could potentially contribute to the deregulation of cell polarity, and should be studied in more detail in the future.

Cortactin is well known as major cellular F-actin binding protein and class-II NPF (Schnoor et al., 2018; Weaver, 2008). Because of these central cellular properties, cortactin also represents a favorite target of various microbial pathogens by promoting their adhesion, invasion and intracellular trafficking (Schnoor et al., 2018; Weaver, 2008). For instance, the gastric pathogen and carcinogen *H. pylori* can exploit cortactin activity in order to control cell attachment to the extracellular matrix and cell motility using its translocated effector protein CagA (Sharafutdinov et al., 2022b; Tegtmeyer et al., 2011). In addition, previous studies revealed that ectopically expressed CagA binds Par1b to induce its inactivation and disrupt cell polarity (Saadat et al., 2007). Here, we found that *H. pylori* infection effectively promotes CagA/Par1b/ZO-1 interactions with cortactin (Fig. 8C). In particular, we discovered this CagA/Par1b/ZO-1/cortactin complex in the TJs (Fig. 8D) and in extruding cells (Fig. S7/S8). This pathway is different to the previously described CagA-ASPP2 interaction, which targets the above-described aPKC-containing regulatory complex (Buti et al., 2020). Thus, we are referring to these two CagA-mediated Par1b-targeting signaling complexes as complex-1 and complex-2, respectively (Fig. 8D). Remarkably, our IP experiments have shown that ASPP2 is absent from complex-1 (Figs. 5B and 7D/E). Thus, there are clearly two distinct signaling pathways, by which *H. pylori* CagA can affect Par1b function (Fig. 8D).

Using specific expression constructs and IP experiments, complex-1 composed of Par1b/cortactin/ZO-1 could be reconstituted in cells. We propose two models of how these proteins could interact with each other and CagA (Fig. 8D and S9). We identified the cortactin phosphorylation sites at positions S405 and S418 to be important for establishing interactions with both Par1b and ZO-1. The main regulatory activities of cortactin during *H. pylori* infection appear through phosphorylation events on serines or tyrosines (Schnoor et al., 2018). Injection of CagA leads to cortactin serine phosphorylation at residues S405 or S418, which is beneficial for binding and activation of N-WASP (Martinez-Quiles et al., 2004) and FAK (Tegtmeyer et al., 2011), established via interactions of their proline-rich regions [PxxP] with the SH3 domain of cortactin. It now appears that phosphorylation at S405 and S418 is also a prerequisite for the interaction of cortactin with Par1b and ZO-1. We found that deletion of the cortactin SH3 domain abrogated its interaction with Par1b/ZO-1, confirming that the SH3 domain is the interaction site. Direct cortactin/Par1b interactions should not be excluded, as Par1b possesses three conserved [PxxP] motifs that could potentially interact with the SH3 domain of cortactin. The first proline-rich motif [PAIP] of Par1b (isoform d) corresponds to residues 415–418, and the two PASP-motifs to amino acids 454–457 and 617–620 within the Par1b spacer domain (Fig. S10). However, cortactin rather employs its SH3 domain for interacting with the PRR of ZO-1. A model in which CagA, upon delivery, interacts with the catalytic domain of Par1b leading to its inactivation and relocation to the apicolateral side of the host cell was proposed, but its exact localization has hitherto remained unknown (Saadat et al., 2007). In TJs, the SH3 domain of ZO-1 could potentially bind one of the yet unknown PxxP motifs of the Par1b spacer domain. Finally, if the cortactin SH3 domain indeed interacted with the PRR of ZO-1 (Katsube et al., 1998), it could form a complex with CagA/Par1b, resulting in cytoskeletal rearrangements and loss of cell polarity. We propose the G**P**AI**P** site of Par1b (Fig. S10) to be a candidate SH3-interacting X**P**xX**P** motif, wherein X typically corresponds to a hydrophobic residue and x is any residue (Aitio et al., 2010). Similar X**P**xX**P** proline-rich motifs A**P**AA**P** and P**P**QI**P** were previously found in dynamin-2 and appeared to be important for interactions with the SH3 domain of cortactin (McNiven et al., 2000).

The loss of epithelial cell polarity and disruption of cell-cell connections by *H. pylori* may have a crucial role in inducing carcinogenesis (Royer & Lu, 2011). In this regard, *H. pylori* CagA represents an established factor promoting gastric carcinogenesis (Hatakeyama, 2014). Cortactin seems to link the pro-oncogenic activity of CagA with actin-cytoskeletal rearrangements, by deregulation of Par1b and cellular ZO-1 functions in the TJs. Cortactin overexpression has been linked to the promotion of carcinogenesis in various organs, including gastric cancer (Wei et al., 2014). In addition, we have previously shown that *H. pylori* CagA can induce cortactin overexpression in a JNK-dependent manner (Sharafutdinov et al., 2021). Furthermore, *H. pylori* expresses the serine protease HtrA, which triggers serious epithelial damage by cleavage of the junctional proteins occludin, claudin-8 and E-cadherin, and it increases the delivery of CagA and other virulence responses, together causing malignant transformation of target cells (Sharafutdinov et al., 2022a, Sharafutdinov et al., 2023; Tegtmeyer et al., 2017). Collectively, these and previous data point to an important role of cortactin in the promotion of CagA-mediated disruption of cellular junctions and cell polarity. Our study was primarily conducted using the polarized Caco-2 cell line of intestinal origin, and we have seen some differences compared to gastric mucosoids. In particular, we observed more prominent Par1b staining in the apical compartments of non-infected gastric mucosoids as compared to non-infected Caco-2 monolayers. These differences could be explained by the mucus layer produced in gastric mucosoids, which is less pronounced in Caco-2 cells (Jantaree et al., 2022; Navabi et al., 2013). Mucin production was shown to be connected to cell polarity, which could potentially affect Par1b distribution closer to the apical side (Kufe, 2009). In our experiments, *H. pylori* infection led to the redistribution of Par1b from basal to apical sides in both Caco-2 cell monolayers and gastric mucosoids (Fig. 6A–B; Fig. 7B–C). Future investigations employing gastric organoid models and/or human gastric sections will provide further insights into the role of cortactin in *H. pylori*-driven gastric diseases. A better understanding of the molecular mechanisms involved in the pathogenesis of *H. pylori* is anticipated to help in developing novel strategies to prevent gastric diseases including gastric cancer.

## 4 Materials and methods

### 4.1 Cultivation of cell lines

Human colon adenocarcinoma Caco-2 (ATCC HTB-37) and human gastric adenocarcinoma AGS (ATCC CRL-1739) cell lines were grown in DMEM or RPMI 1640 (Gibco, Darmstadt, Germany) media, respectively, supplemented with 10% fetal calf serum (Gibco), 1% penicillin/streptomycin (Sigma-Aldrich, Steinheim, Germany), and 0.2% normocin (InvivoGen, Toulouse, France). For experiments, cells were routinely cultured in 6-well or 12-well plates until they reached confluency. 16 h prior to infection, the culture medium was replaced with an antibiotic-free medium. All cell lines were recently authenticated by German Collection of Microorganisms and Cell Cultures GmbH (DMSZ, Braunschweig, Germany) and are routinely tested for exclusion of mycoplasma contamination once a month.

### 4.2 Cultivation, infection and analysis of mucosoids

Human gastric organoids (GAT23) were derived from antral gastric tissue samples from Charite University Medicine Berlin (EA1/129/12). Gastric organoids cultured on collagen-coated inserts of transwell chambers lead to polarized epithelial layers (mucosoid cultures) that secrete mucus on the apical surface. The long-term stable mucosoid cultures were passaged every three weeks and express the gastric stem cell marker Lgr5 and markers for differentiated gland phenotypes including pepsinogen C for chief cells, MUC6 for cells at the base of the gland and MUC5AC for foveolar cells. In contrast, expression of ATP4B, which is characteristic of parietal corpus cells, was absent. Mucosoid cultures between passages 5–20 were used for experiments. Gastric mucosoids were cultured as described previously (Jantaree et al., 2022). In brief, 4 × 10^5^ cells derived from gastric organoids were seeded onto a collagen-coated (15 μg/cm^2^, A10644-01, Gibco®) membrane of a 12-well ThinCert™ insert. The cells were cultivated in a mucosoid culture medium (advance DMEM/F12, 12634-010, Thermo Fisher Scientific) and 25% (v/v) R-Spondin conditioned medium supplemented with 25 ​ng/ml Wnt Surrogate-Fc Fusion Protein (N001, U-Protein Express B.V.), 2% (v/v) B27™ Supplement (50x) (17504-044, Thermo Fisher Scientific), 10 ​mM Nicotinamide (N0636-100G, Sigma Aldrich), 1 % (v/v) Penicillin-Streptomycin (100x) (15140-122, Thermo Fisher Scientific), 1% (v/v) N-2 Supplement (100x) (17502-048, Thermo Fisher Scientific), 20 ​ng/ml Human EGF (PHG0311, Thermo Fisher Scientific), 1 ​μM TGF-β RI Kinase Inhibitor IV (Alk-I) (616454, Calbiochem), 150 ​ng/ml Human FGF-10 (100-26, PeproTech), 150 ​ng/ml Human Noggin (120-10C, PeproTech), 10 ​nM Human [Leu^15^]-Gastrin I (G9145, Sigma Aldrich) and 7.5 μM ROCK inhibitor (Y-27632) (Y0503, Sigma Aldrich) at 37 °C in a humidified 5 % CO_2_ atmosphere. The medium over the cells was removed on day 4 after seeding to create an air-liquid interface (ALI). The medium at the basolateral side was then replaced with a mucosoid culture medium supplemented with 1.5 μM ROCK inhibitor twice a week. Under ALI culture conditions, the mucosoids produce and accumulate mucous on the apical side, which was removed twice a week. The gastric mucosoids were cultivated for 18 days to form a monolayer with complete barrier integrity.

The mucous was removed before starting the *H. pylori* infection. *H. pylori* strains P1 wt, P1Δ*cagA* and P1Δ*virB7* (Backert et al., 2000) were grown on GC agar plates supplemented with 10% horse serum (Gibco®/Life Technologies), 5 μg/ml trimethoprim (Sigma Aldrich), 1 μg/ml nystatin (Sigma Aldrich), 10 μg/ml vancomycin (*H. pylori* P1wt) (Sigma Aldrich) and 6 μg/ml chloramphenicol (P1Δ*cagA*, P1Δv*irB7*) (Sigma Aldrich) under microaerophilic conditions at 37 °C for 48 h prior to infection. Cells were infected with *H. pylori* at multiplicity of infection (MOI) 100 for 6 h. Prior to analysis, the mucosoid cultures on the membrane of the ThinCert™ inserts were washed once with PBS w/o Ca^2+^/Mg^2+^ (14190-094, Thermo Fisher Scientific), and fixed with 4% paraformaldehyde for 15 min at room temperature.

### 4.3 Inactivation of cortactin in Caco-2 cells by CRISPR/Cas9

The cortactin gene *cttn* was inactivated in Caco-2 cells by application of CRISPR/Cas9 technology. Constitutive exons within the *cttn* gene were disrupted by transfection of the Cortactin CRISPR/Cas9 KO (human, sc-400761, Santa Cruz Heidelberg, Germany) and three Cortactin HDR (human, sc-400761-HDR) plasmids. The three Cortactin CRISPR/Cas9 KO plasmids A, B and C each encode Cas9 nuclease as well as a target-specific 20-nucleotide guide RNA directed against exons 3 and 12 that encode the NTA and F-actin binding domains of cortactin, respectively. The three Cortactin HDR plasmids A, B and C were co-transfected with Cortactin CRISPR/Cas9 KO plasmids in order to repair the site-specific Cas9-induced DNA breaks within the *cttn* gene. During co-transfection, the HDR plasmid conferred a puromycin resistance cassette and expression of red fluorescent protein (RFP), enabling selection of stable knockout (KO) cells and confirmation of successful transfection by fluorescence microscopy. After transfection, cells were selected in medium supplemented with 2 μg/mL puromycin. RFP-expressing, single cells were sorted using fluorescence activated cell sorting (FACS Aria II SORP; BD Bioscience, Heidelberg, Germany) and checked for viability by fluoresence microscopy. The cells were grown in a 96-well plate (Greiner Bio-One, Frickenhausen, Germany) until reaching confluence, after which they were gradually transferred into larger plates. Western blotting and immunofluorescence microscopy using cortactin-specific antibodies were employed to confirm *cttn* gene disruption.

### 4.4 *H. pylori* strains and culture conditions

*H. pylori* wt strains N6, P1, P12, 26695, and the isogenic mutants N6Δ*cagA*, N6Δ*virB10*, P1Δ*cagA*, P1Δ*cagA*/*cagA*^wt^, P1Δ*cagA*/*cagA*^YF^, P1Δ*cagA*/*cagA*^ΔCRPIA^, P1Δ*cagA*/*cagA*^FLAG^, and P1Δ*cagY*Δ*cagA*/*cagA*^FLAG^ were routinely generated as described (Backert et al., 2001) and stored in stocks at −80 °C (BHI medium containing 20% glycerol). For infection experiments, bacteria were freshly taken from the stocks and grown on GC agar plates supplemented with 10% horse serum, 10 μg/mL vancomycin, and 4 μg/mL amphotericin. Bacteria were grown in a 2.5 L anaerobic jar (Oxoid, Wesel, Germany) with a CampyGen (Oxoid) package to provide microaerophilic conditions at 37 °C.

### 4.5 Transepithelial electrical resistance

To study transepithelial electrical resistance (TEER) of Caco-2 wt and Caco-2Δ*cttn* cells, the cells were grown in the transwell system with filter inserts of 3 μm pore size (Corning B.V. Lifescience, Schiphol, Netherlands). Briefly, 1 × 10^5^ Caco-2 wt or Caco-2Δ*cttn* cells were seeded onto the filter inserts in 12-well plates and grown until reaching full confluence. TEER measurements were performed using the Electrical Resistance System (ERS) (Merck Millipore, Burlington, MA, USA). During cell growth, TEER was measured every second to third day followed by a medium change. The obtained TEER values were adjusted considering medium resistance and area of inserts, and expressed as Ohms/cm^2^.

### 4.6 *H. pylori* transepithelial migration

In order to study the transmigration rate of *H. pylori* through the polarized Caco-2 wt and Caco-2Δ*cttn* epithelial cell monolayers, cells were infected with bacteria from the apical side with an MOI of 50. Samples of medium with transmigrated bacteria were taken from the basal compartment of the transwell filter system, and then plated out on Mueller-Hinton agar plates. The plated bacteria were incubated for 4–6 days under microaerophilic conditions at 37 °C, 5% CO_2_, followed by counting of the colony-forming units (CFUs).

### 4.7 Transfection studies

AGS cell lines expressing cortactin, wild-type cortactin S405D, cortactin S418D, cortactin Y421D, cortactin Y466D, cortactinΔSH3, cortactinΔSH3/ΔPRR or Par1b were generated by transfection of the cells with 4 μg pEGFP-C1-Cortactin, pEGFP-C1-Cortactin S405D, pEGFP-C1-Cortactin S418D, pEGFP-C1-Cortactin Y421D, pEGFP-C1-Cortactin Y466D, pEGFP-C1-CortactinΔSH3, pEGFP-C1-CortactinΔSH3/ΔPRR (Tegtmeyer et al., 2011) or pEF-His-Par1b wt (gift of M. Hatakeyama, M.D., Ph.D., Department of Microbiology, Graduate School of Medicine, University of Tokyo, Tokyo, Japan) using the transfection reagent Turbofect according to manufacturer's instructions (Thermo Fisher Scientific). Following a defined incubation, the cells were either infected with bacteria and processed further for immunoblotting, or used for immunoprecipitations.

### 4.8 Immunoprecipitation (IP)

First, Caco 2 wt cells were infected with *H. pylori* for a defined time as indicated in figure legends. After infections, cells were prepared for IPs as described earlier (Tegtmeyer et al., 2011). The precipitated protein complexes were analyzed by immunoblotting.

### 4.9 Immunoblot analysis

Proteins in cell lysates or from IPs were separated in conventional SDS-PAGE gels followed by transfer onto PVDF membranes for Western blotting. Membranes were blocked with either 5% non-fat milk or 3% BSA and probed with the primary antibodies as follows: mouse α-cortactin (#05–180, Merck-Millipore), rabbit α-Par1b (#HPA074905, Sigma Aldrich), rabbit α-ZO-1 (#61–73000, Invitrogen), rabbit α-E-cadherin (#sc-7870, Santa Cruz), rabbit α-Claudin-5 (#ab15106, Abcam), mouse α-β-actin (#A5441, Sigma Aldrich), mouse α-GFP (#632381, Clontech), rabbit α-omni-probe (α-T7) (#sc-499, Santa Cruz), rabbit α-CagA (#HPP-5003-9, Austral Biologicals), mouse α-PY99 (#sc-7020, Santa Cruz) and α-ASPP2 (#200-401-A19, Rockland). After washing with TBS-T, membranes were incubated with the respective α-mouse or α-rabbit secondary antibodies conjugated with horseradish peroxidase (Thermo Fisher Scientific). The antibodies were detected using the ECL Plus chemiluminescence Western Blot kit (GE Healthcare Life Sciences).

### 4.10 Fluorescence microscopy

Caco-2 wt or Caco-2Δ*cttn* cells were grown on coverslips or in transparent transwell cell inserts in 12-well plates as described above. Human mucosoids were grown on transparent transwell cell inserts. After reaching full confluence, cells were infected with *H. pylori* as indicated or left untreated. Afterwards, cells were fixed with 4% PFA at RT for 10 min and permeabilized with either 0.25% Triton X-100 or 0.1% Saponin. For immunostaining, primary mouse α-cortactin (#05–180, Merck-Millipore), rabbit α-Par1b (#HPA074905, Sigma Aldrich), rabbit α-ZO-1 (#61–73000, Invitrogen), mouse α-FLAG (#F3165, Sigma Aldrich), mouse α-occludin (#33–1511, Invitrogen) or mouse α-E-cadherin (#612130, BD Biosciences) antibodies were used. Secondary reagents were α-mouse FITC-conjugated, α-mouse TRITC-conjugated as well as α-rabbit AlexaFluor-633-conjugated antibodies. Optionally, cells were counterstained with TRITC-conjugated phalloidin (#R415, Invitrogen) or 4′-6-diamidino-2-phenylindole dihydrochloride (DAPI, Invitrogen) for the detection of the F-actin cytoskeleton or cell nuclei, respectively. The samples were further mounted onto glass slides, dried and analyzed with an epifluorescence microscope, Leica DMI4000B (Leica Microsystems, Wetzlar, Germany) or by using a confocal laser scanning microscope, Leica Stellaris 8 (Leica Microsystems, Wetzlar, Germany). In order to analyze Caco-2 cell morphology, samples were imaged by light microscopy using phase contrast optics. Cortactin and HDR plasmid in Caco-2 or Caco-2Δ*cttn* cells were detected using green and red filters of the fluorescence microscope, respectively. The LAS AF computer software (Leica Microsystems, Wetzlar, Germany) was used to process obtained raw data. Further analyses of microscopy images including quantification of cell/nuclei areas, quantification of cellular tight junction length, distribution of fluorescence across monolayers from basal to apical direction (Z-profiles), distribution of fluorescence across X-axis within given X/Z area (X-profile) were all performed by processing the microscopy images using ImageJ software (Schindelin et al., 2012).

### 4.11 Scanning electron microscopy

Caco-2 wt or Caco-2Δ*cttn* cells were grown on glass cover slips (12 mm diameter) until they reached confluence. The cells were then fixed for 1 h at 4 °C in HEPES buffer (0.1 M HEPES, 0.09 M sucrose, 0.01 M CaCl_2_, 0.01 M MgCl_2_, pH 6.9) including 5% formaldehyde and 2% glutaraldehyde, and washed twice with TE buffer (10 mM Tris, 2 mM EDTA). Afterwards, dehydration of the samples was performed using a graded series of acetone (10%, 30%, 50%, 70%, 90%, and 100%) for 10 min at each step at RT. The automated CPD300 dryer (Leica Microsystems, Wetzlar, Germany) was used to carry out critical point drying. The cover slips were then mounted on 12 mm aluminum stubs with Leit adhesive carbon tabs and sputter-coated with gold/palladium in the SCD 500 (Bal-Tec, Balzers, Lichtenstein). To analyze the samples, a Merlin field emission scanning electron microscope (Zeiss, Oberkochen, Germany) was employed at an acceleration voltage of 5 kV, an inlens-Se detector/Everhart-Thornley SE detector ratio of 75:25, and with optimized contrast and brightness settings.

### 4.12 Bioinformatics

Domain architecture of Par1b/MARK2 kinase (isoform d) was predicted by using the NCBI conserved domain database (CDD) search (Wang et al., 2023). In order to identify the putative proline-rich motifs [PxxP] within Par1b, the protein sequence of Par1b/MARK2 isoform d (NP 001034558.2) was used for blastp searches for similar proteins within mammals (taxid: 40674) in the NCBI RefSeq Protein database. Further alignments and searches for conserved motifs were performed by using MUSCLE (multiple sequence comparison by log-expectation) algorithm in Mega X (Edgar, 2004; Kumar et al., 2018).

### 4.13 Statistical analyses

All experiments were at least done in three biological replicates with similar results. The cells were infected and randomly analyzed in a blinded fashion by different members of the team. All data were evaluated using GraphPad Prism as statistical software (Version 8.0, GraphPad Software Inc., San Diego, CA, USA). Two-tailed *t*-test was used to compare the means of two groups. One-way ANOVA with Tukey's multiple comparison test was used for the comparison of more than two groups. The TEER values in Caco-2 wt and Caco-2Δ*cttn* cells lines obtained in a time-course were analyzed by the two-way ANOVA analysis with two independent variables (cell line vs growth time). Statistical significance was defined as p ​≤ ​0.05 (∗), p ​≤ ​0.01 (∗∗) and p ​≤ ​0.001 (∗∗∗), ns – not significant.

## Data availability

This study includes no data deposited in external repositories. All inquiries for any reagents used in this report should be sent to Steffen Backert (steffen.backert@fau.de).

## Author contributions

SB and NT conceptualized the study. IS conducted the confocal imaging studies, bioinformatics and statistics. AH generated the cortactin knockout clones and transfected the cells. NT performed the infections, IPs and Western blotting. MM conducted the scanning EM studies, and CT and MN the mucosoid analyses. HS performed protein-protein interaction modeling. SB, IS and KR analyzed the data and wrote the manuscript. All co-authors reviewed and edited the final paper.

## Declaration of competing interest

The authors declare that they have no known competing financial interests or personal relationships that could have appeared to influence the work reported in this paper.

## References

[bib1] Aitio O., Hellman M., Kazlauskas A., Vingadassalom D.F., Leong J.M., Saksela K., Permi P. (2010). Recognition of tandem PxxP motifs as a unique Src homology 3-binding mode triggers pathogen-driven actin assembly. Proceedings of the National Academy of Sciences of the United States of America.

[bib2] Amieva M.R., Vogelmann R., Covacci A., Tompkins L.S., Nelson W.J., Falkow S. (2003). Disruption of the epithelial apical-junctional complex by *Helicobacter pylori* CagA. Science.

[bib3] Ammer A.G., Weed S.A. (2008). Cortactin branches out: Roles in regulating protrusive actin dynamics. Cell Motility and the Cytoskeleton.

[bib4] Böhm H., Brinkmann V., Drab M., Henske A., Kurzchalia T.V. (1997). Mammalian homologues of C-elegans PAR-1 are asymmetrically localized in epithelial cells and may influence their polarity. Current Biology.

[bib5] Backert S., Muller E.C., Jungblut P.R., Meyer T.F. (2001). Tyrosine phosphorylation patterns and size modification of the *Helicobacter pylori* CagA protein after translocation into gastric epithelial cells. Proteomics.

[bib6] Backert S., Neddermann M., Maubach G., Naumann M. (2016). Pathogenesis of *Helicobacter pylori* infection. Helicobacter.

[bib7] Backert S., Ziska E., Brinkmann V., Zimny-Arndt U., Fauconnier A., Jungblut P.R., Naumann M., Meyer T.F. (2000). Translocation of the *Helicobacter pylori* CagA protein in gastric epithelial cells by a type IV secretion apparatus. Cellular Microbiology.

[bib8] Benton R., St Johnston D. (2003). *Drosophila* PAR-1 and 14-3-3 inhibit Bazooka/PAR-3 to establish complementary cortical domains in polarized cells. Cell.

[bib9] Buckley C.E., St Johnston D. (2022). Apical-basal polarity and the control of epithelial form and function. Nature Reviews Molecular Cell Biology.

[bib10] Buti L., Ruiz-Puig C., Sangberg D., Leissing T.M., Brewer R.C., Owen R.P., Sgromo B., Royer C., Ebner D., Lu X. (2020). CagA-ASPP2 complex mediates loss of cell polarity and favors *H. pylori* colonization of human gastric organoids. Proceedings of the National Academy of Sciences of the United States of America.

[bib11] Buti L., Spooner E., Van der Veen A.G., Rappuoli R., Covacci A., Ploegh H.L. (2011). *Helicobacter pylori* cytotoxin-associated gene A (CagA) subverts the apoptosis-stimulating protein of p53 (ASPP2) tumor suppressor pathway of the host. Proceedings of the National Academy of Sciences of the United States of America.

[bib12] Cao L., Ghasemi F., Way M., Jégou A., Romet-Lemonne G. (2023). Regulation of branched versus linear Arp2/3-generated actin filaments. EMBO Journal.

[bib13] Cao L., Way M. (2024). The stabilization of Arp2/3 complex generated actin filaments. Biochemical Society Transactions.

[bib14] Citalan-Madrid A.F., Vargas-Robles H., Garcia-Ponce A., Shibayama M., Betanzos A., Nava P., Salinas-Lara C., Rottner K., Mennigen R., Schnoor M. (2017). Cortactin deficiency causes increased RhoA/ROCK1-dependent actomyosin contractility, intestinal epithelial barrier dysfunction, and disproportionately severe DSS-induced colitis. Mucosal Immunology.

[bib15] Edgar R.C. (2004). Muscle: Multiple sequence alignment with high accuracy and high throughput. Nucleic Acids Research.

[bib16] Fischer W., Tegtmeyer N., Stingl K., Backert S. (2020). Four Chromosomal type IV secretion systems in *Helicobacter pylori*: Composition, structure and function. Frontiers in Microbiology.

[bib17] Fregoso F.E., Boczkowska M., Rebowski G., Carman P.J., van Eeuwen T., Dominguez R. (2023). Mechanism of synergistic activation of Arp2/3 complex by cortactin and WASP-family proteins. Nature Communications.

[bib18] Gautreau A.M., Fregoso F.E., Simanov G., Dominguez R. (2022). Nucleation, stabilization, and disassembly of branched actin networks. Trends in Cell Biology.

[bib19] Grainger C.I., Greenwell L.L., Lockley D.J., Martin G.P., Forbes B. (2006). Culture of Calu-3 cells at the air interface provides a representative model of the airway epithelial barrier. Pharmaceutical Research.

[bib20] Guo S., Sokolova O.S., Chung J., Padrick S., Gelles J., Goode B.L. (2018). Abp1 promotes Arp2/3 complex-dependent actin nucleation and stabilizes branch junctions by antagonizing GMF. Nature Communications.

[bib21] Hatakeyama M. (2014). *Helicobacter pylori* CagA and gastric cancer: A paradigm for hit-and-run carcinogenesis. Cell Host Microbe.

[bib22] Hirakawa H., Shibata K., Nakayama T. (2009). Localization of cortactin is associated with colorectal cancer development. International Journal of Oncology.

[bib23] Jantaree P., Yu Y.F., Chaithongyot S., Tager C., Sarabi M.A., Meyer T.F., Boccellato F., Maubach G., Naumann M. (2022). Human gastric fibroblasts ameliorate A20-dependent cell survival in co-cultured gastric epithelial cells infected by *Helicobacter pylori*. Biochimica Et Biophysica Acta-Molecular Cell Research.

[bib24] Javaheri A., Kruse T., Moonens K., Mejias-Luque R., Debraekeleer A., Asche C.I., Tegtmeyer N., Kalali B., Bach N.C., Sieber S.A. (2017). *Helicobacter pylori* adhesin HopQ engages in a virulence-enhancing interaction with human CEACAMs. Nature Microbiology.

[bib25] Katsube T., Takahisa M., Ueda R., Hashimoto N., Kobayashi M., Togashi S. (1998). Cortactin associates with the cell-cell junction protein ZO-1 in both Drosophila and mouse. Journal of Biological Chemistry.

[bib26] Knorr J., Sharafutdinov I., Fiedler F., Esmaeili S.D., Rohde M., Rottner K., Tegtmeyer N. (2021). Cortactin is required for efficient Fak, Src and Abl tyrosine kinase activation and phosphorylation of *Helicobacter pylori* CagA. International Journal of Molecular Sciences.

[bib27] Krueger S., Hundertmark T., Kuester D., Kalinski T., Peitz U., Roessner A. (2007). *Helicobacter pylori* alters the distribution of ZO-1 and p120ctn in primary human gastric epithelial cells. Pathology, Research & Practice.

[bib28] Kufe D.W. (2009). Mucins in cancer: Function, prognosis and therapy. Nature Reviews Cancer.

[bib29] Kumar S., Stecher G., Li M., Knyaz C., Tamura K. (2018). Mega X: Molecular evolutionary genetics analysis across computing platforms. Molecular Biology and Evolution.

[bib30] Kwok T., Zabler D., Urman S., Rohde M., Hartig R., Wessler S., Misselwitz R., Berger J., Sewald N., Konig W. (2007). *Helicobacter* exploits integrin for type IV secretion and kinase activation. Nature.

[bib31] Liu T., Cao L., Mladenov M., Jegou A., Way M., Moores C.A. (2024). Cortactin stabilizes actin branches by bridging activated Arp2/3 to its nucleated actin filament. Nature Structural & Molecular Biology.

[bib32] Martinez-Quiles N., Ho H.Y.H., Kirschner M.W., Ramesh N., Geha R.S. (2004). Erk/Src phosphorylation of cortactin acts as a switch on-switch off mechanism that controls its ability to activate N-WASP. Molecular and Cellular Biology.

[bib33] McNiven M.A., Kim L., Krueger E.W., Orth J.D., Cao H., Wong T.W. (2000). Regulated interactions between dynamin and the actin-binding protein cortactin modulate cell shape. The Journal of Cell Biology.

[bib34] Moonens K., Hamway Y., Neddermann M., Reschke M., Tegtmeyer N., Kruse T., Kammerer R., Mejias-Luque R., Singer B.B., Backert S. (2018). *Helicobacter pylori* adhesin HopQ disrupts trans dimerization in human CEACAMs. The EMBO journal.

[bib35] Moujaber O., Stochaj U. (2020). The cytoskeleton as regulator of cell signaling pathways. Trends in Biochemical Sciences.

[bib36] Navabi N., McGuckin M.A., Lindén S.K. (2013). Gastrointestinal cell lines form polarized epithelia with an adherent mucus layer when cultured in semi-wet interfaces with mechanical stimulation. PLoS One.

[bib37] Ohsawa S., Vaughen J., Igaki T. (2018). Cell extrusion: A stress-responsive force for good or evil in epithelial homeostasis. Developmental Cell.

[bib38] Piontek J., Krug S.M., Protze J., Krause G., Fromm M. (2020). Molecular architecture and assembly of the tight junction backbone. Biochimica Et Biophysica Acta-Biomembranes.

[bib39] Royer C., Lu X. (2011). Epithelial cell polarity: A major gatekeeper against cancer?. Cell Death & Differentiation.

[bib40] Ruch T.R., Engel J.N. (2017). Targeting the mucosal barrier: How pathogens modulate the cellular polarity network. Cold Spring Harbor Perspectives in Biology.

[bib41] Saadat I., Higashi H., Obuse C., Umeda M., Murata-Kamiya N., Saito Y., Lu H.S., Ohnishi N., Azuma T., Suzuki A. (2007). *Helicobacter pylori* CagA targets PAR1/MARK kinase to disrupt epithelial cell polarity. Nature.

[bib42] Salama N.R., Hartung M.L., Muller A. (2013). Life in the human stomach: Persistence strategies of the bacterial pathogen *Helicobacter pylori*. Nature Reviews Microbiology.

[bib43] Schindelin J., Arganda-Carreras I., Frise E., Kaynig V., Longair M., Pietzsch T., Preibisch S., Rueden C., Saalfeld S., Schmid B. (2012). Fiji: An open-source platform for biological-image analysis. Nature Methods.

[bib44] Schnoor M., Stradal T.E., Rottner K. (2018). Cortactin: Cell functions of A multifaceted actin-binding protein. Trends in Cell Biology.

[bib45] Selbach M., Backert S. (2005). Cortactin: An achilles' heel of the actin cytoskeleton targeted by pathogens. Trends in Microbiology.

[bib46] Selbach M., Moese S., Hauck C.R., Meyer T.F., Backert S. (2002). Src is the kinase of the *Helicobacter pylori* CagA protein *in vitro* and *in vivo*. Journal of Biological Chemistry.

[bib47] Selbach M., Moese S., Hurwitz R., Hauck C.R., Meyer T.F., Backert S. (2003). The *Helicobacter pylori* CagA protein induces cortactin dephosphorylation and actin rearrangement by c-Src inactivation. EMBO Journal.

[bib48] Sharafutdinov I., Backert S., Tegtmeyer N. (2020). Cortactin: A major cellular target of the gastric carcinogen *Helicobacter pylori*. Cancers.

[bib49] Sharafutdinov I., Backert S., Tegtmeyer N. (2021). The *Helicobacter pylori* type IV secretion system upregulates epithelial cortactin expression by a CagA- and JNK-dependent pathway. Cellular Microbiology.

[bib50] Sharafutdinov I., Ekici A., Vieth M., Backert S., Linz B. (2022). Early and late genome-wide gastric epithelial transcriptome response during infection with the human carcinogen *Helicobacter pylori*. Cell Insight.

[bib51] Sharafutdinov I., Knorr J., Esmaeili D.S., Backert S., Tegtmeyer N. (2022). Cortactin promotes effective AGS cell scattering by *Helicobacter pylori* CagA, but not cellular vacuolization and apoptosis induced by the vacuolating cytotoxin VacA. Pathogens.

[bib52] Sharafutdinov I., Knorr J., Rottner K., Backert S., Tegtmeyer N. (2022). Cortactin: A universal host cytoskeletal target of gram-negative and gram-positive bacterial pathogens. Molecular Microbiology.

[bib53] Sharafutdinov I., Tegtmeyer N., Linz B., Rohde M., Vieth M., Tay A.C.-Y., Sticht H. (2023). A single-nucleotide polymorphism in *Helicobacter pylori* promotes gastric cancer development. Cell Host Microbe.

[bib54] Tegtmeyer N., Harrer A., Rottner K., Backert S. (2021). *Helicobacter pylori* CagA induces cortactin Y-470 phosphorylation-dependent gastric epithelial cell scattering via Abl, Vav2 and Rac1 activation. Cancers.

[bib55] Tegtmeyer N., Wessler S., Necchi V., Rohde M., Harrer A., Rau T.T., Figueiredo C. (2017). *Helicobacter pylori* employs a unique basolateral type IV secretion mechanism for CagA delivery. Cell Host Microbe.

[bib56] Tegtmeyer N., Wittelsberger R., Hartig R., Wessler S., Martinez-Quiles N., Backert S. (2011). Serine phosphorylation of cortactin controls focal adhesion kinase activity and cell scattering induced by *Helicobacter pylori*. Cell Host Microbe.

[bib57] Tokuda S., Furuse M. (2015). Claudin-2 knockout by TALEN-mediated gene targeting in MDCK cells: Claudin-2 independently determines the leaky property of tight junctions in MDCK cells. PLoS One.

[bib58] Wang J.Y., Chitsaz F., Derbyshire M.K., Gonzales N.R., Gwadz M., Lu S.N., Marchler G.H., Song J.S., Thanki N., Yamashita R.A. (2023). The conserved domain database in 2023. Nucleic Acids Research.

[bib59] Weaver A.M. (2008). Cortactin in tumor invasiveness. Cancer Letters.

[bib60] Wei J., Zhao Z.X., Li Y., Zhou Z.Q., You T.G. (2014). Cortactin expression confers a more malignant phenotype to gastric cancer SGC-7901 cells. World Journal of Gastroenterology.

[bib61] Yin M., Ma W.Q., An L.G. (2017). Cortactin in cancer cell migration and invasion. Oncotarget.

[bib62] Zakrzewski S.S., Richter J.F., Krug S.M., Jebautzke B., Lee I.F.M., Rieger J., Sachtleben M., Bondzio A., Schulzke J.D., Fromm M. (2013). Improved cell line IPEC-J2, characterized as a model for porcine jejunal epithelium. PLoS One.

[bib63] Zihni C., Mills C., Matter K., Balda M.S. (2016). Tight junctions: From simple barriers to multifunctional molecular gates. Nature Reviews Molecular Cell Biology.

