# Supplementary material for: Cortactin-dependent control of Par1b-regulated epithelial cell polarity in Helicobacter infection
Source: Cell Insight. 2024 Mar 5;3(3):100161. doi: 10.1016/j.cellin.2024.100161 (PMC11033139; doi:10.1016/j.cellin.2024.100161)
Supplement: Multimedia component 1 [file mmc1.docx]

**Supplementary Material**

**
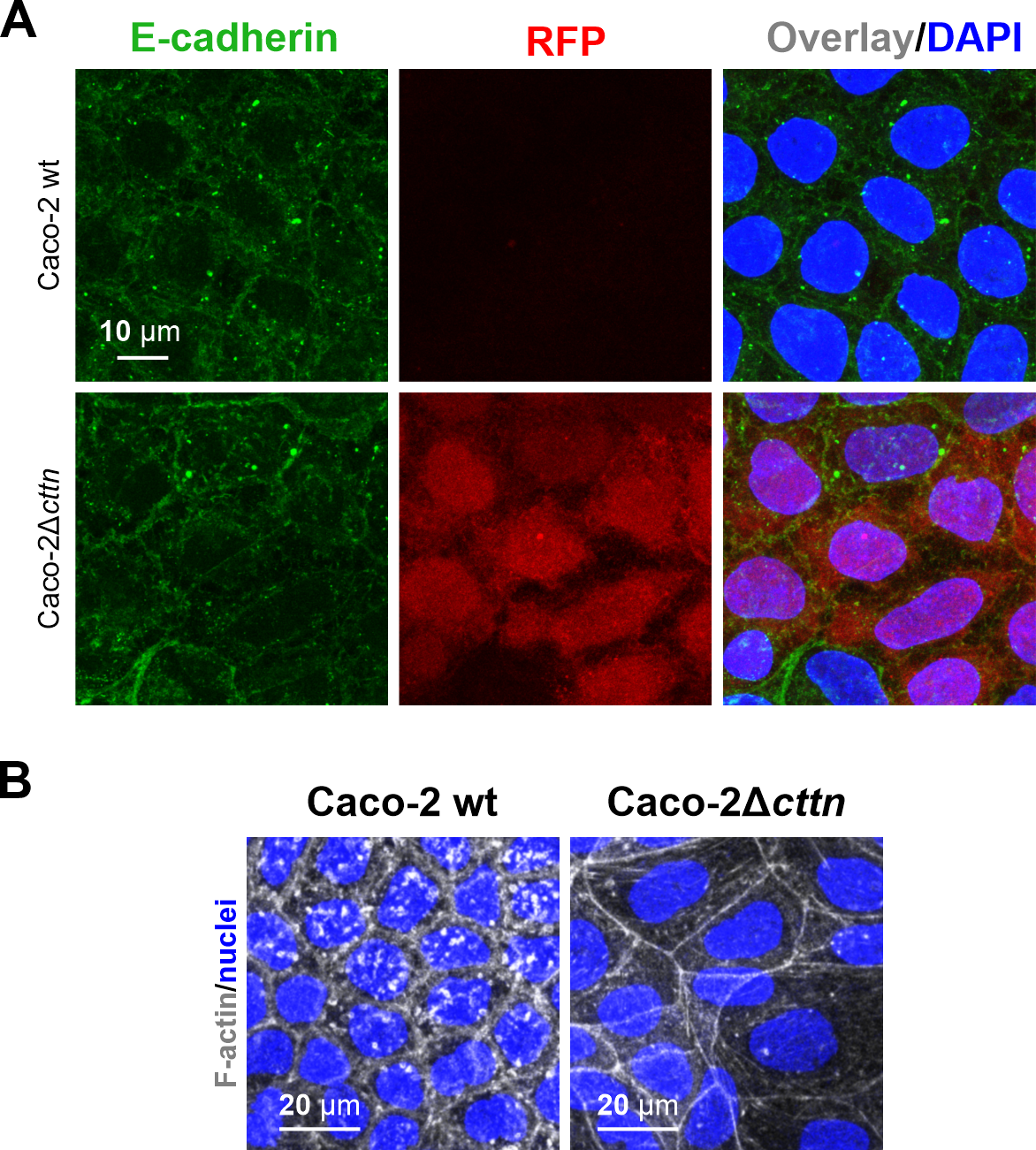
**

**Figure S1.** (**A**) Fluorescence microscopy of Caco-2 wt and Caco-2Δ*cttn* cells immunostained with E-cadherin-specific antibodies. Expression of RFP from the functional HDR plasmid (red) served as a control. (**B**) Caco-2 wt or Δ*cttn* monolayers were stained with phalloidin and DAPI to visualize F-actin (white) and cell nuclei (blue), respectively. Further, fluorescence microscopy images were used for the analysis of cellular and nuclear areas.

**
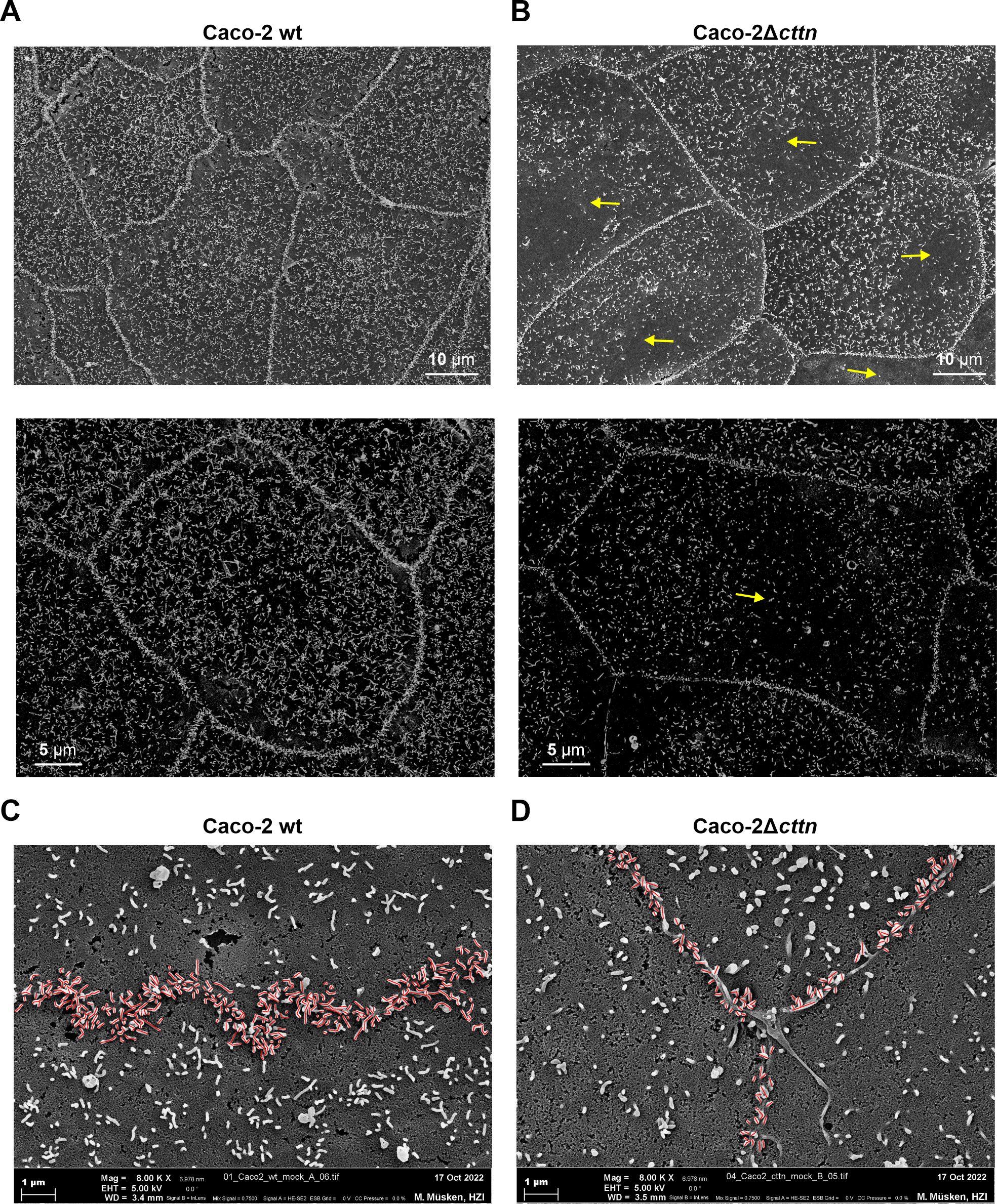
**

**Figure S2**. Scanning electron microscopy (SEM) of further examples of (**A**) Caco-2 wt and (**B**) Caco-2Δ*cttn* monolayers showing microvilli distribution across the cells. The cell with reduced development of microvilli is indicated by the yellow arrow. Representative areas of (**C**) Caco-2 wt and (**D**) Caco-2Δ*cttn* monolayers showing cellular junctions with microvilli. The microvilli number and their length were quantified as indicated by the red coloring.

**
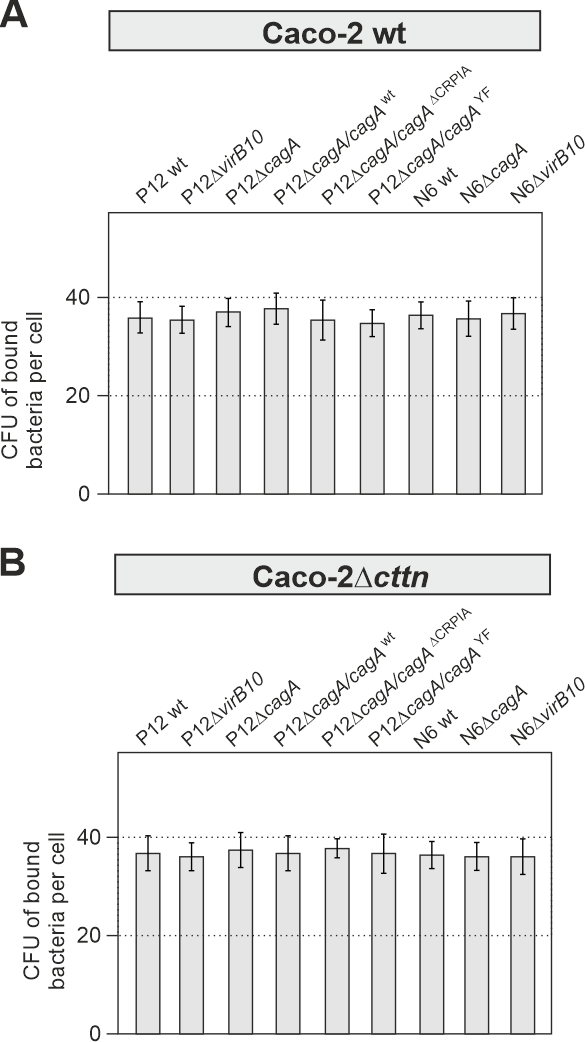
**

**Figure S3**. Caco-2 wt (**A**) or Caco-2Δ*cttn* (**B**) were infected with various *H. pylori* strains used in this study, and the number of adherent *H. pylori* was quantified afterwards by determining the colony forming units (CFU).

**
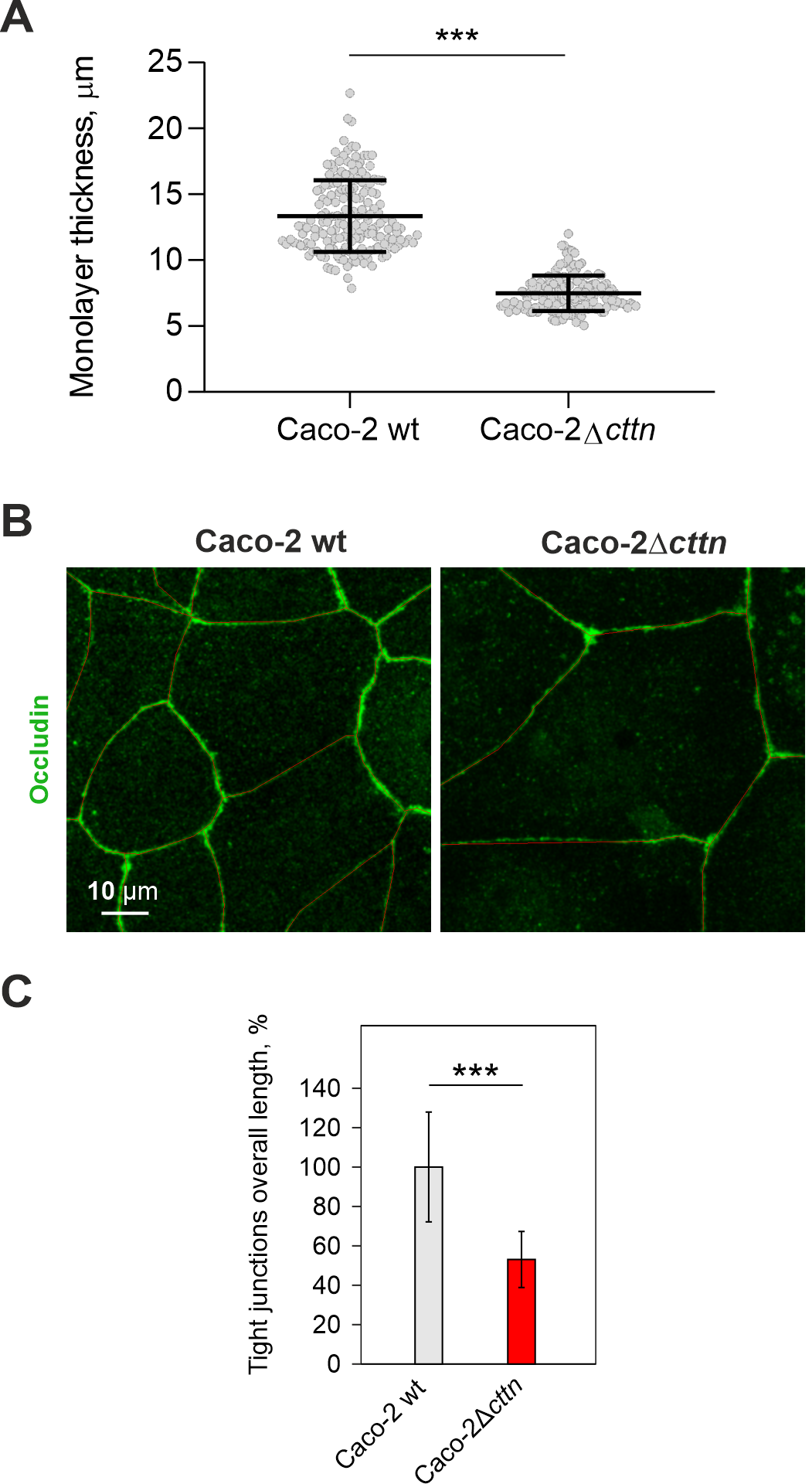
**

**Figure S4**. (**A**) Thickness of Caco-2 wt and Caco-2Δ*cttn* cells across the monolayers; p<0.001 (***). (**B**) Representative micrographs of Caco-2 wt and Caco-2Δ*cttn* monolayers showing occludin immunostaining (green), and used for the analysis of the overall length of tight junctions within an image. (**C**) The overall length of tight junctions in Caco-2 wt and Caco-2Δ*cttn* monolayers was measured per micrograph, and the mean value per micrograph in Caco-2 wt cells was set to 100%; p<0.001 (***).


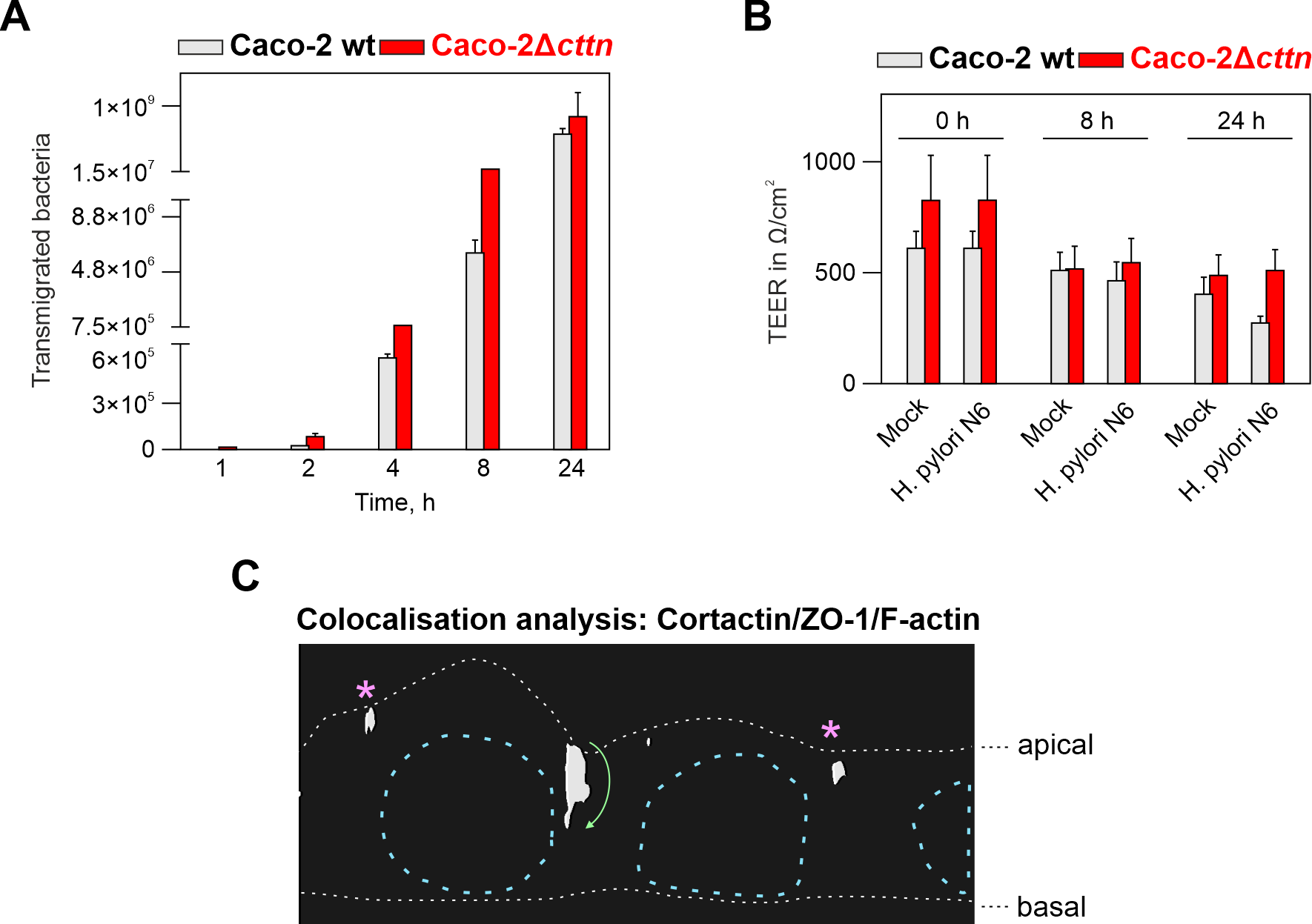


**Figure S5.** (**A**) The number of *H. pylori* cells, transmigrated through Caco-2 wt or Caco-2Δ*cttn* cell monolayers was assessed in a time course using transwell filter systems. (**B**) The TEER dynamics due to infection time was measured. (**C**) Colocalization analysis of cortactin, ZO-1 and F-actin in a Caco-2 monolayer after infection with *H. pylori* wt. The white spots within monolayers represent the areas where the fluorescence of all proteins was detected. Cortactin and F-actin colocalized along with ZO-1 at both apical (purple stars) and apicolateral sides (green arrow). Blue dashed circles schematically represent cell nuclei.

**
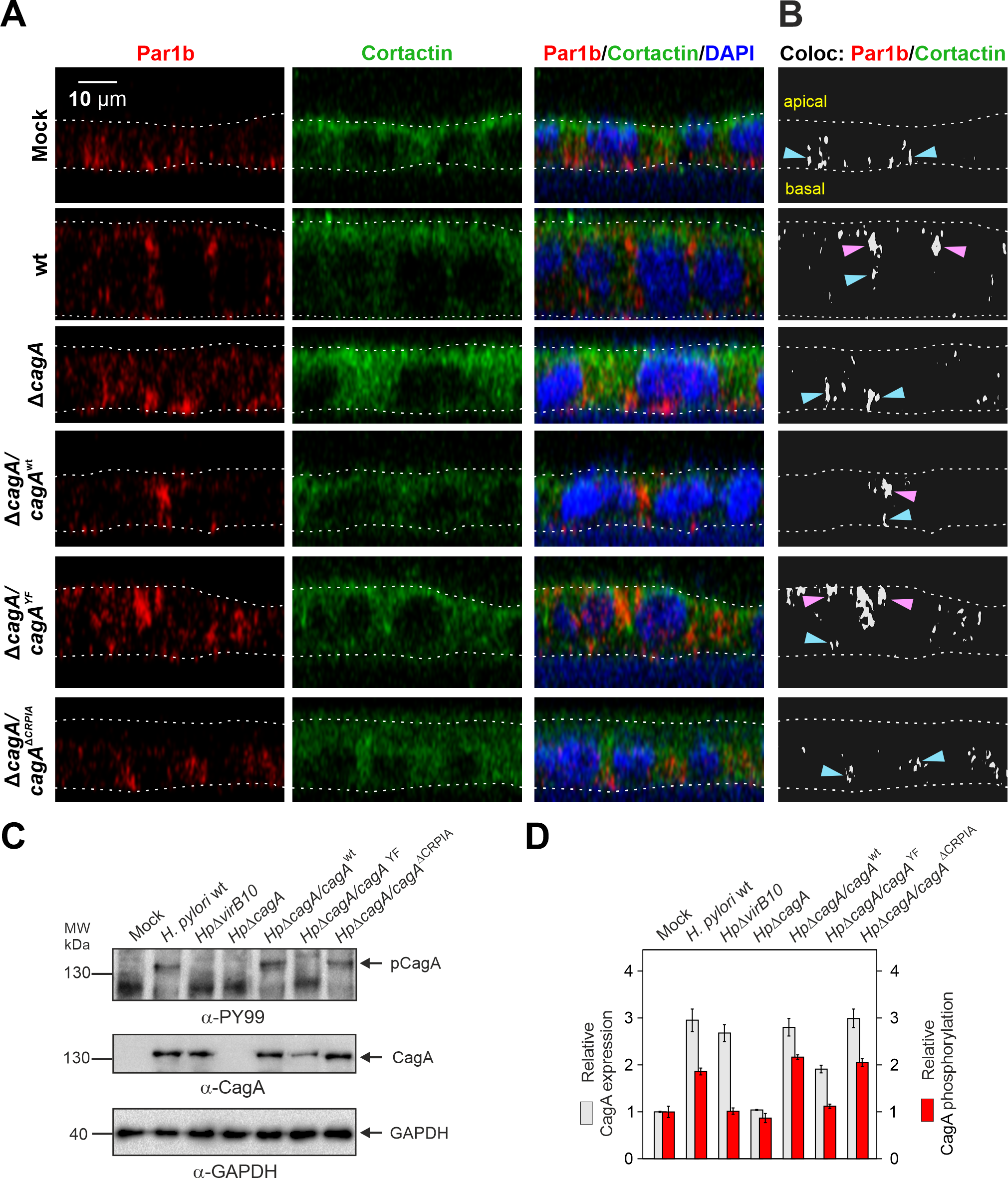
**

**Figure S6**. **A**) Fluorescence microscopy of Caco-2 cell monolayer cross-sections (X/Z dimension) without or after infection with indicated *H. pylori* strains. The white dashed lines indicate apical and basal surfaces of the monolayers. Monolayers were immunostained for Par1b (red) and cortactin (green), and counterstained for nuclei (blue). (**B**) Co-localization analysis of Par1b and cortactin. The white spots within monolayers represent the areas where the fluorescence of both proteins was detected. Pink and blue arrowheads indicate mislocalized apicolateral and normal basolateral protein distribution, respectively. (**C**) Infected AGS cells served as control for proper infection, documented by Western blotting using antibodies against phosphorylated and non-phosphorylated CagA. Tyrosine-phosphorylated CagA indicated successful infection. (**D**) The relative CagA expression and phosphorylation were quantified, respectively; the mean intensities ± SDs are presented.


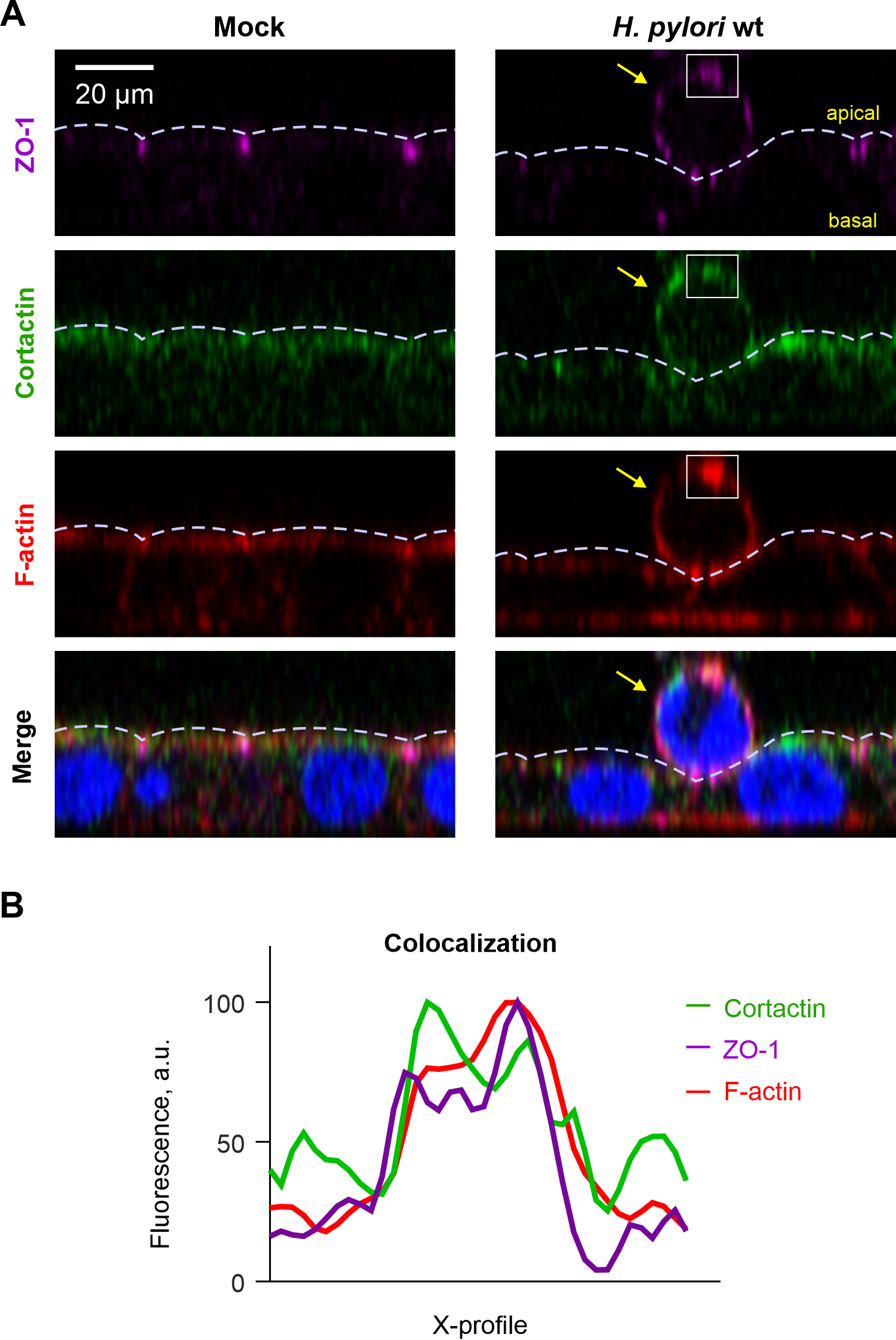


**Figure S7**. (**A**) Representative Caco-2 cell extruding from the epithelial monolayer (indicated by yellow arrows) without or after infection with *H. pylori*. Cell monolayers were immunostained for cortactin (green) and ZO-1 (violet) and counterstained for F-actin (red) and nuclei (blue). (**B**) Colocalization analysis of cortactin, ZO-1 and F-actin in the cell area indicated by white rectangles in panel **A**. The relative fluorescence intensities of the proteins were plotted against their spatial distribution across X-axis within the indicated X/Z-area (X-profile); a.u. – arbitrary units.

**
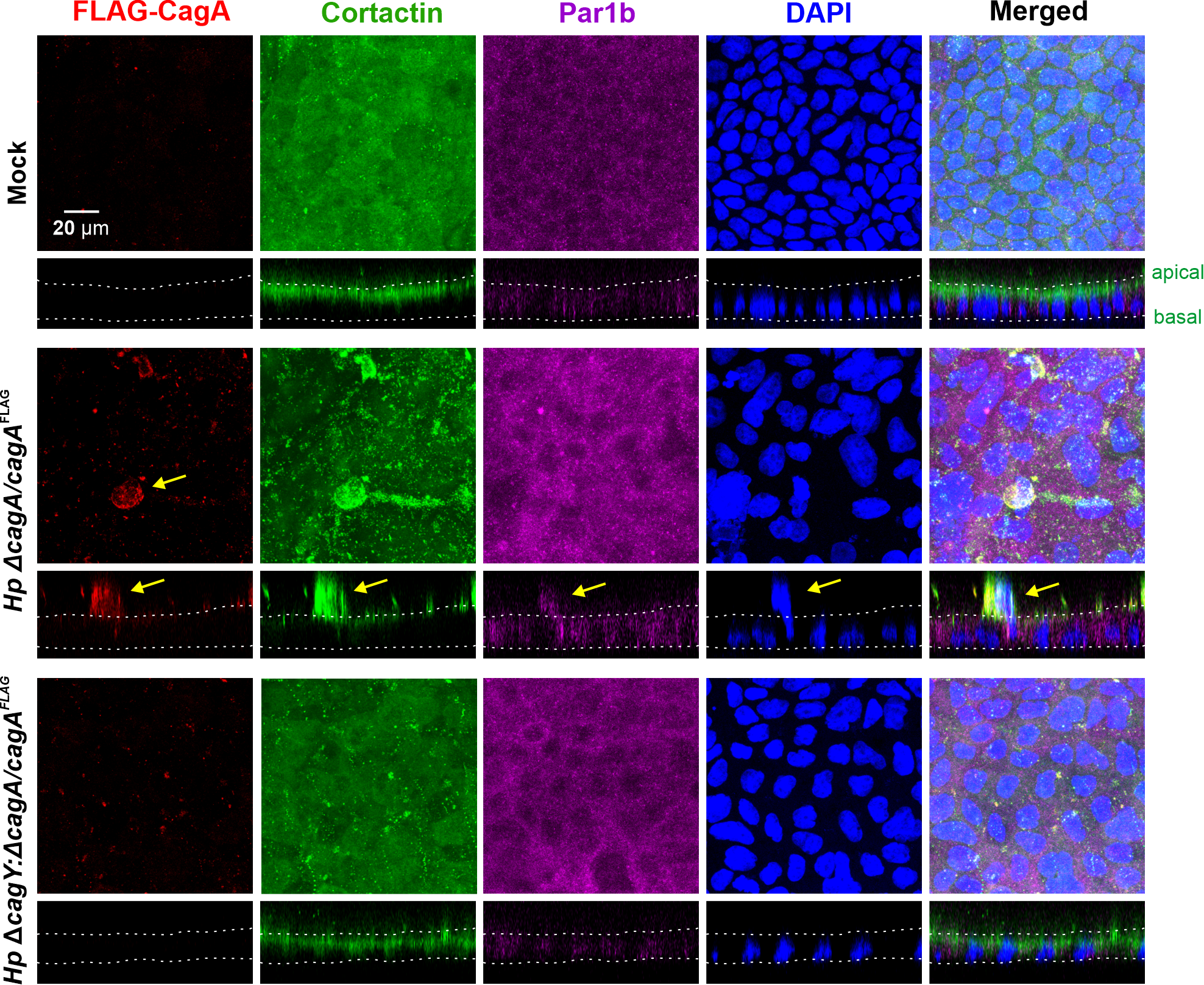
**

**Figure S8**. Fluorescence microscopy of Caco-2 cell monolayers (top view and cross-sections) after infection with *H. pylori* wt or Δ*virB10* mutant possessing FLAG-tagged CagA. Monolayers were stained for FLAG (red), cortactin (green), Par1b (violet), and nuclei (blue). Yellow arrow indicates the epithelial cell enriched with CagA-FLAG, cortactin and Par1b, and extruding from the monolayer. The white dashed lines indicate apical and basal surfaces of the monolayer.

**
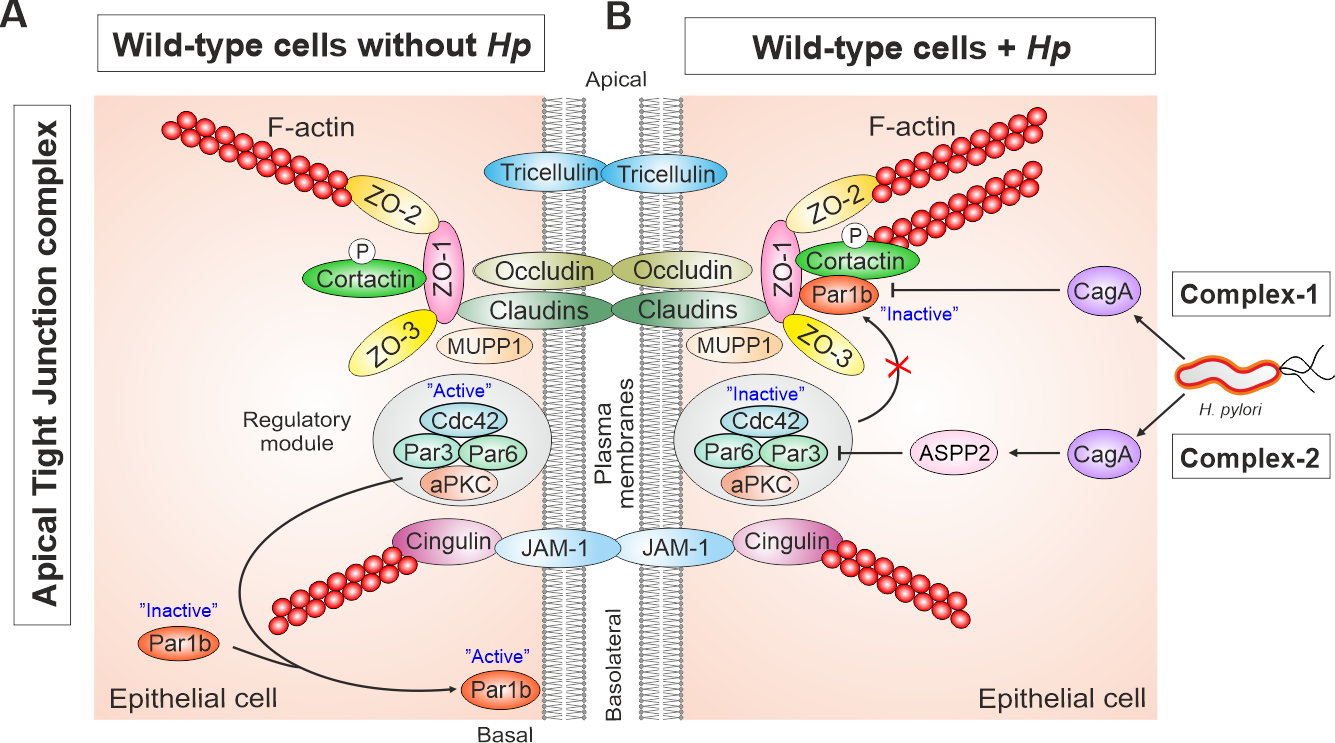
**

**Figure S9**. Scheme showing the detailed composition of the apical TJ complex in epithelial cells before and after *H. pylori* infection. (**A**) In non-infected cells, various important proteins and their position in the TJ complex are highlighted, including cortactin binding to ZO-1. The active regulatory complex composed of aPKC/Par3/Par6/Cdc42 phosphorylates partitioning kinase Par1b (active form), which maintains the apical-junctional and basal polarities of the cell. This regulatory complex and basal Par1b are mutually antagonistic, which altogether ensures cell polarity. (**B**) During infection with *H. pylori,* CagA is injected into epithelial cells, which targets Par1b and cell polarity by two different pathways, here named complex-1 and complex-2 (for more details see the main Fig. 8D).

**
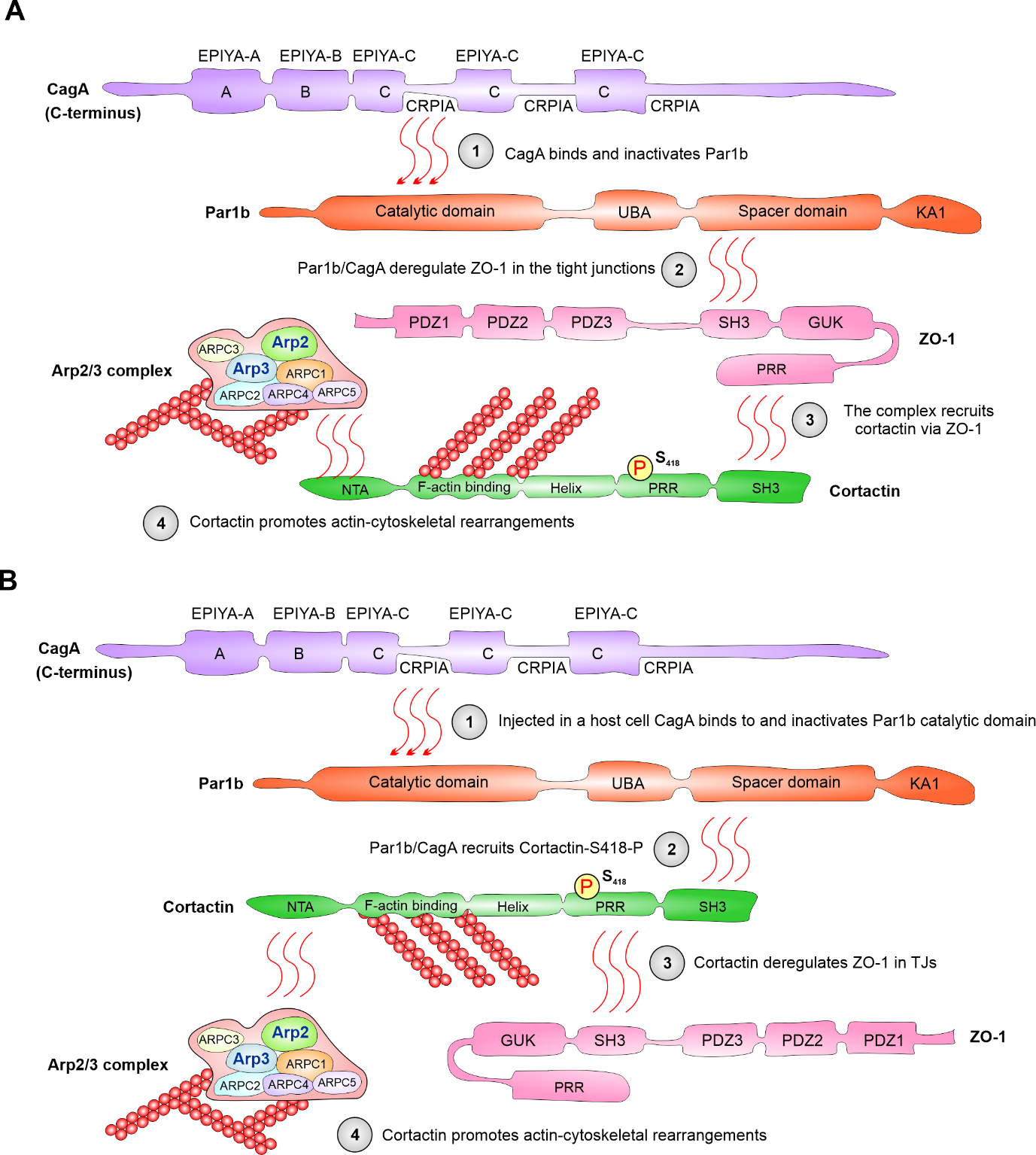
**

**Figure S10**. Schematic models of proposed interaction events of cortactin, CagA, Par1b and ZO-1 at the domain level. Both models assume that the CagA/Par1b complex forms first. (**A)** This complex can either interact with ZO-1 via putative Par1b-PRR/ZO-2-SH3 domain interaction. Then, cortactin phosphorylated at S418 is recruited to ZO-1 in the TJs by cortactin- SH3/ZO-1-PRR domain interactions. (**B**) Alternatively, the CagA/Par1b complex can interact with cortactin via putative Par1b-PRR/cortactin-SH3 domain interactions. Then, cortactin phosphorylated at S418 is recruited to ZO-1 in the TJs by cortactin-PRR/ZO-1-SH3 domain interactions. We consider both protein complexes of CagA/Par1b/ZO-1/cortactin as reasonable. This established complex then triggers a change in cell polarity and actin-cytoskeletal rearrangements, e.g. via Arp2/3 complex and F-actin filament binding as indicated.

**
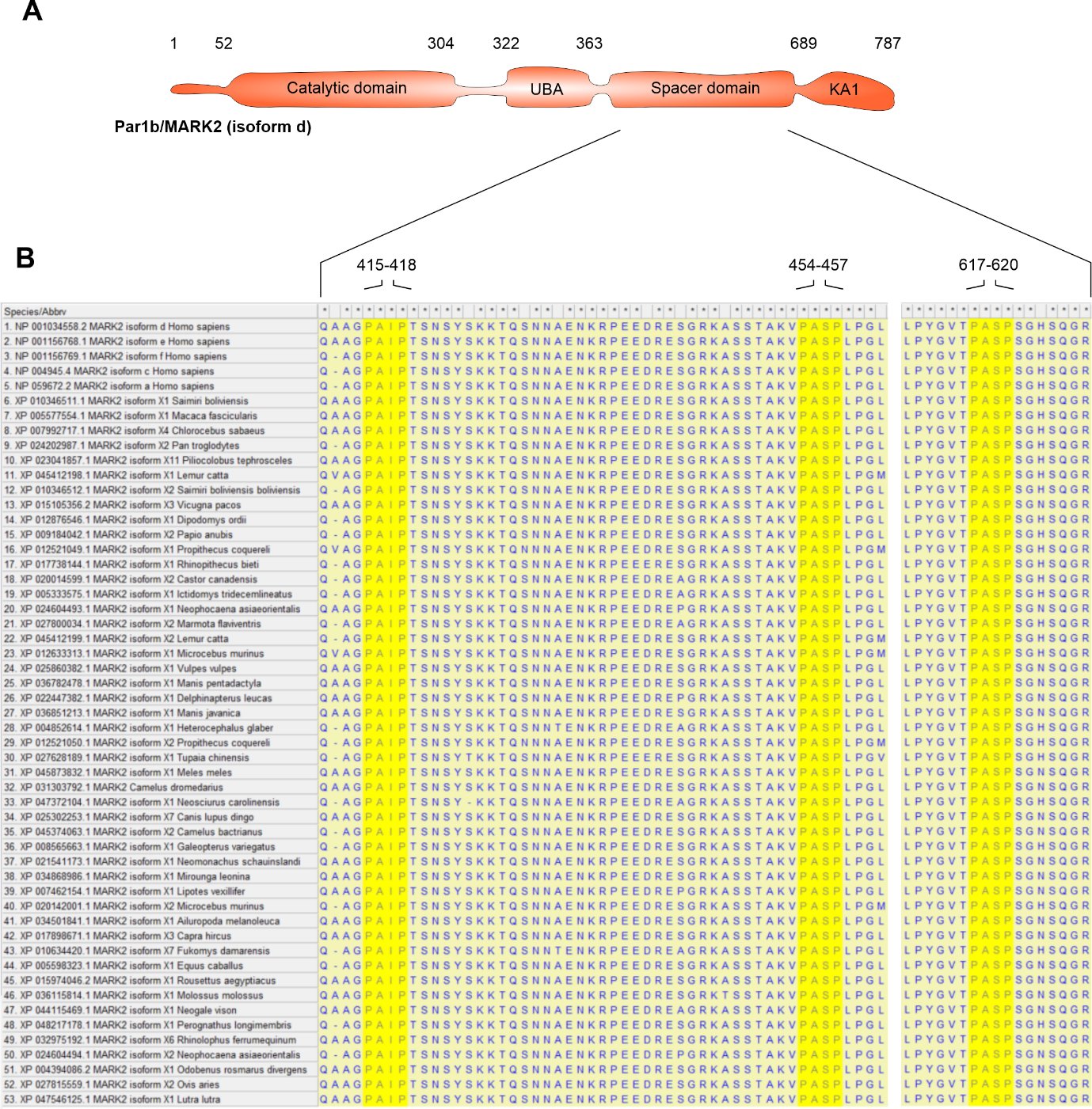
**

**Figure S11**. (**A**) Domain architecture of Par1b/MARK2 kinase (isoform d) as predicted by the NCBI conserved domain database (CDD) search. (**B**) Putative proline-rich motifs [PxxP] of Par1b/MARK2 isoform d (NP 001034558.2) aligned against reference proteins within mammals (taxid: 40674). The proline-rich motif PAIP at position 415-418 preceded by glycine is a putative interaction site with an SH3 domain that is present in both ZO-1 and cortactin.
